# Zinc/iron-regulated transporter-like protein gene family in *Theobroma cacao* L: Characteristics, evolution, function and 3D structure analysis

**DOI:** 10.3389/fpls.2023.1098401

**Published:** 2023-02-28

**Authors:** Daniel Dastan Rezabala Pacheco, Brenda Conceição Guimaraes Santana, Carlos Priminho Pirovani, Alex-Alan Furtado de Almeida

**Affiliations:** Department of Biological Sciences, Santa Cruz State University, Campus Soane Nazaré, Ilhéus, Bahia, Brazil

**Keywords:** *Theobroma cacao*, ZIP gene family, metal transport, phylogenetics, chromosomal location, homology modeling, PPI network

## Abstract

The zinc/iron-regulated transporter-like protein (ZIP) gene family first identified in plants is highly distributed in the plant kingdom. This family has previously been reported to transport several essential and non-essential cationic elements, including those toxic to many economically important crops such as cacao (*Theobroma cacao* L.). In this article, we present a detailed study on physicochemical properties, evolution, duplication, gene structure, promoter region and TcZIP family three-dimensional protein structure. A total of 11 TcZIP genes have been identified to encode proteins from 309 to 435 aa, with localization in the plasma membrane and chloroplast, containing 6-9 putative domains (TM). Interspecies phylogenetic analysis subdivided the ZIP proteins into four groups. Segmental duplication events significantly contributed to the expansion of TcZIP genes. These genes underwent high pressure of purifying selection. The three-dimensional structure of the proteins showed that α helix conformations are predominant with several pocket sites, containing the metal binding site, with the residues leucine (LEU), alanine (ALA), glycine (GLY), serine (SER), lysine (LYS) and histidine (HIS) the most predicted. Regarding the analysis of the protein-protein interaction and enrichment of the gene ontology, four biological processes were assigned, the most important being the cation transport. These new discoveries expand the knowledge about the function, evolution, protein structures and interaction of ZIP family proteins in cacao and contribute to develop cacao genotypes enriched with important mineral nutrients as well as genotypes that bioaccumulate or exclude toxic metals.

## 1 Introduction

Zinc (Zn) and iron (Fe) are key elements for plant growth and development. (Zn) is an enzymatic cofactor, participating in electron transport and antioxidant metabolism. On the other hand, it is an integral element of several transcription factors and plays important roles in enzyme activation, gene expression, protein synthesis, photosynthesis and carbohydrate metabolism (Chang et al., 2005; Slaton et al., 2005). (Fe) is required for electron transport in chloroplasts and mitochondria. It is involved in the hormones biosynthesis and integrity of cellular organelles; as well as in the tolerance to oxidative stress and in the N fixation (Hänsch and Mendel, 2009). Zn and Fe deficiency causes many nutritional, biochemical and molecular disorders. A complex control system allows the uptake, distribution and accumulation of these ions, ensuring metal homeostasis. The uptake of metal ions, under deficiency conditions, activates several transport proteins, such as the zinc/iron-regulated transporter-like protein (ZIP) family. Nowadays, members of the ZIP family have been identified in bacteria, archaea, and eukaryotes as well as protists, animal fungi, and plants (Mäser et al., 2001). ZIP gene family in the model species *Arabidopsis thaliana* has been extensively studied. For most ZIP proteins, the topology with the N-terminal and C-terminal ends is predicted to be located on the outside surface of the plasma membrane, with eight potential transmembrane domains (TMDs). The variable region contains a potential metal-binding domain rich in histidine residues (Guerinot, 2000).

Studies on the function of ZIP genes in *A. thaliana* and the uptake of metal ions start with the IRTI (iron-regulated transporter) gene, which was the first identified gene encoding a transporter involved in the Fe^2+^ uptake with localization in the plasma membrane (Eide et al., 1996). IRT3 and ZIP4 genes are induced by Zn deficiency and are involved in the Zn increase in xylem (Grotz et al., 1998). IRT1 is also responsible for the uptake of elements such as cadmium (Cd) and Zn. Plants with overexpression of IRT1 hold higher levels of these elements (Connolly et al., 2002). Genes expressed in roots and leaves such as ZIP2, ZIP4, ZIP5 and ZIP9 are up-regulated in Zn-deficient conditions, while IRT2 is involved in Fe uptake. Two, ZIP2 and ZIP4 genes are also transcriptionally regulated by copper (Cu) (Wintz et al., 2003). Other members of this family, such as ZIP1 and ZIP3, are mostly expressed in roots and are implicated in Zn uptake in *A. thaliana* (Ishimaru et al., 2005). IRT1, a gene which plays a role in the Fe, manganese (Mn), Zn and Cd uptake (Cohen et al., 1998; Vert et al., 2002) also mediates the accumulation of nickel (Ni) in *A. thaliana* (Nishida et al., 2011). After that, a lot of ZIP family members have been found in the species *Oriza sativa* (Ishimaru et al., 2011). The genes OsZIP1, OsZIP4, OsZIP5 and OsZIP8 are involved in the Zn transport and distribution. The localization of these proteins is in the plasma membrane of roots and shoots (Ishimaru et al., 2007; Lee et al., 2010; Lee et al., 2010; Yang et al., 2009). Thus the ZIP plays key roles in the uptake and translocation of essential and non-essential metal ions in different plant organs (Eide et al., 1996; Grotz et al., 1998; Wintz et al., 2003; Li et al., 2013; Milner et al., 2013) and has been studied in several plant species of economic importance, such as *Zea mays* (Li et al., 2013), *Glycine max* (Moreau et al., 2002), *Hordeum vulgare* (Tiong et al., 2014), *Vitis vinífera* (Gainza-Cortés et al., 2012), *Solanum tuberosum* (Li et al., 2020) and *Poncirus trifoliata* (Fu et al., 2017).

Information on ZIPs was not systematically analyzed in the *T. cacao* genome, hampering the understanding of its role in the processes of uptake and distribution of essential and non-essential elements such as Cd, which is toxic and commonly affects this crop (Araújo et al., 2017). *T. cacao* genome, at the chromosomal level, in its recent high-quality version (Motamayor et al., 2013) provides quality resources to study, at the genomic level, the role of the ZIP gene family.

In the present study, we were the first to identify and characterize members of the ZIP gene family based on phylogenetic relationships, physico-chemical properties, transmembrane domains, conserved motifs, exon/intron organization, substitution rates, chromosomal locations and cis-regulatory elements in cacao. We also explored post-translational modifications, 3D modeling, prediction of ZIP transport protein pocket sites and protein-protein interaction (PPI) network. The results reported in the present study will increase our knowledge about the evolution, function, structure and interaction of ZIPs, being the first step towards future studies on the processes of uptake and translocation of essential and non-essential elements in cacao.

## 2 Materials and methods

### 2.1 Identification and annotation of ZIP family members in the genome of *T. cacao*

We retrieved the Zn/Fe-regulated transporter-like protein (ZIP) family protein sequences from The *Arabidopsis* Information Resource database (https://www.arabidopsis.org/). The ZIP genes in cacao (*Theobroma cacao*) were identified for the first time using the Protein-protein Basic Local Alignment Search Tool (BLASTP) (Altschul et al., 1990) with cut-off point set to 1e^-4^ (Duan et al., 2016). As a query sequence, we used all known *A. thaliana* ZIP family proteins against the latest version of the *T. cacao* genome obtained from the Phytozome database (https://phytozome-next.jgi.doe.gov/). All putative hits were examined using Pfam database (Punta et al., 2012) to verify the presence or absence of the conserved ZIP domain (PF02535). To confirm the presence of the ZIP domain the sequences were finally verified using the Simple Modular Architecture Research (SMART) database with a cut-off p-value of 1.0 (Letunic et al., 2012). All non-redundant sequences were used for further analysis. The ZIP family genes identified in cacao were designated TcZIP.

### 2.2 Characterization of the physico-chemical properties of the putative sequences

The physico-chemical properties, concerning protein length, molecular weight (MW), isoelectric point (IP), the grand average of hydropathicity (GRAVY), were determined using the ProtParam tool (http://www.expasy.org/tools/). The subcellular location of the ZIP genes was predicted using the Plant-mPLoc server (Chou and Shen, 2010). Potential transmembrane domains were identified using the TMHMM tool (Krogh et al., 2001).

### 2.3 Phylogenetic relationships, exon-intron gene structures analysis and protein motifs identification

The full sequences of transport proteins ZIPs from *T. cacao* and *A. thaliana* were aligned by the MEGA11 software, using the Clustal W algorithm. According to the alignment results, we build a phylogenetic tree using the Neighbor-Joining (NJ) method as implemented in MEGA11 software (Tamura et al., 2021) with 1000 bootstrap replicates. Furthermore, another phylogenetic tree, comprising all TcZIP proteins, was constructed using the same method. Also we analyzed the sequences of all TcZIP genes to identify exon-intron organizations using the Gene Structure Display Server tool (Hu et al., 2015). To identify conserved protein motifs in ZIP transport proteins, we use the Multiple Em for Motif Elicitation server (MEME) (Bailey et al., 2009).

### 2.4 Promoter region analyses, chromosome mapping and ka/ks ratio estimation

PlantCare (Lescot et al., 2002) was used to study cis-regulatory elements in the 1500 bp promoter regions upstream of transcriptional start sites. The chromosomal positions of the ZIP gene’s were extracted from the Phytozome database. ZIP genes approximate locations were mapped onto cacao chromosomes using MapInspect software ((http://www.softsea.com/download/MapInspect.htm) (accessed on 13 Jun 2022). Synonymous (Ks) and non-synonymous (Ka) replacement rates per site among duplicate pairs were calculated using the browser Ka/Ks Calculation tool (http://services.cbu.uib.no/tools/kaks). Duplicate pair splitting time was estimated using a mutation rate synonymous with λ substitutions per synonymous site per year, according to the equation T = (Ks/2λ (λ = 6.5 × 10^-9^)) × 10^-6^ (Yang et al., 2008; Yuan et al., 2015).

### 2.5 Prediction of TcZIP proteins post-translational modifications

The phosphorylation sites of TcZIP proteins were predicted by the NetPhos 3.1 server (Blom et al., 2004) with a value greater than >0.5. The NetNGlyc 1.0 tool was used to predict N-glycosylation sites with default parameters (Gupta and Brunak, 2002).

### 2.6 Three-dimensional protein modeling, validation and analysis of the pocket sites

The prediction of ZIP proteins 3D structure in cacao was made by homology based on templates predicted by the server SWISS-MODEL (Waterhouse et al., 2018). After selecting the best model, we validate the predicted models through the Ramachandran analysis (Lovell et al., 2003). The pockets prediction in predicted models was performed using CASTp tool (Tian et al., 2018). Finally Discovery Studio Visualizer (BIOVIA, 2015) was used to view the results.

### 2.7 Systems biology

An interaction network was constructed from *A. thaliana* proteins homologous to those identified in *T. cacao*. The interactome analysis was performed using the STRING version 11.5 (http://string-db.org) with the following parameters: meaning of network edges: confidence; (ii) active interaction sources: all; (iii) minimum required interaction score: high confidence (0.700); (iv) maximum number of interactors to show: 1st and 2nd shell is no more than 20 interactions. Cytoscape version 3.9.1 was used to merge and analyze all networks. Gene ontology enrichment analysis was performed for each cluster using BiNGO version 3.0.5 plugin. The modularity and centrality properties (betweenness and node degree) of the network were calculated using the igraph package of the statistical tool R.

## 3 Results

### 3.1 Identification and genomic location of ZIP proteins in *T. cacao* genome

**Figure 1:**
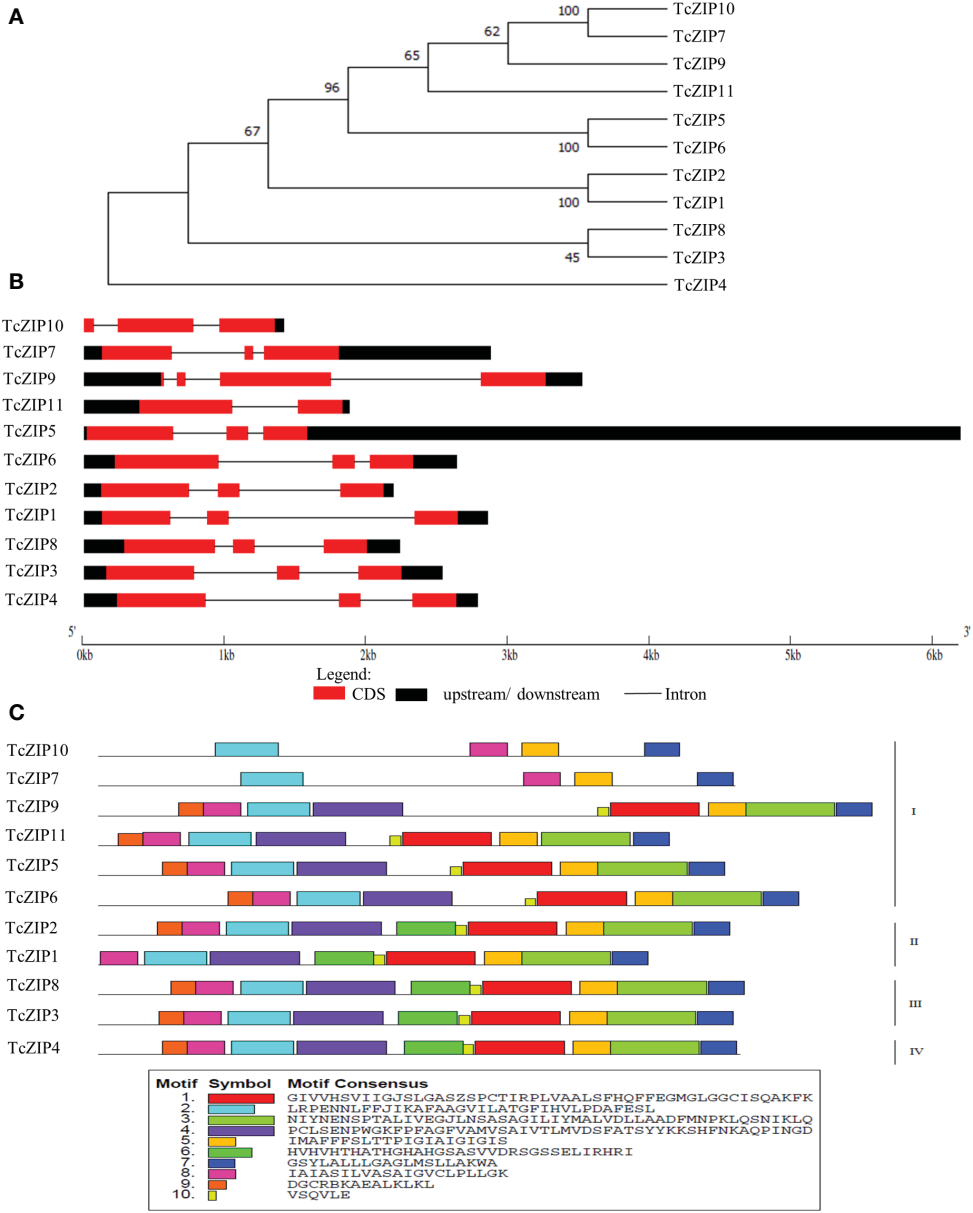
The evolutionary relationships, gene structures and functional motifs of transport proteins in *T. cacao* (TcZIP). **(A)** A phylogenetic tree constructed using MEGA11 software by the NJ method with bootstrap analysis (1000 replicates). **(B)** Gene structures, CDS, upstream/downstream, and introns are shown. **(C)** Composition of each TcZIP proteins motif. Motifs 1-10 are displayed as differently colored boxes with corresponding sequence information for each motif.

After removing the overlapping genes, a total of 11 nonredundant putative genes associated with the Zn/Fe-regulated transporter-like protein (ZIP) gene family in the *T. cacao* genome, were identified and characterized based on their physical-chemical properties (**Table 1**). After that, we designated the TcZIP1 to TcZIP11 genes according to their physical positions on the chromosomes. The 11 genes identified encode proteins containing the ZIP domain. SMART and Pfam analyzes were performed to confirm the ZIP domains that are present in the putative genes in cacao. The ZIP proteins ranged in length from 309 to 435 amino acids, with molecular weights from 32.44 to 46.30 kDa and isoelectric points ranging from 6.03 to 9.02. According to GRAVY values, all ZIP proteins were hydrophobic proteins. Additional analyzes show that the 11 genes contained between 6-9 putative transmembrane domains (TMs), with subcellular localization in the plasma membrane and chloroplasts (**Table 1**). This is consistent with the known characteristics of ZIP genes studied. A phylogenetic tree was constructed with the 11 ZIP transport proteins in cacao, using the Neighbor-Joining (NJ) method. Through these analyses the TcZIP were divided into four groups, with strong bootstrap values (**Figure 1A**).

**Table 1:** Identification of the Zn/Fe-regulated transporter-like protein (ZIP) encoding TcZIP proteins.

Gene	Transcript ID	CDS (bp)	Protein length (aa)	MW (kDa)	Theoretical pI	GRAVY	Subcellular location	Chromosome	TM domain
**TcZIP1**	Thecc.01G173600.1	930	309	32.443	6.73	0.530	Plasma membrane	1	6
**TcZIP2**	Thecc.01G179300.1	1068	355	37.732	6.30	0.449	Plasma membrane	1	7
**TcZIP3**	Thecc.02G004300.1	1074	357	38.193	8.61	0.585	Plasma membrane	2	8
**TcZIP4**	Thecc.02G004400.1	1086	361	38.935	8.09	0.464	Plasma membrane	2	9
**TcZIP5**	Thecc.02G318800.1	1059	352	37.797	7.55	0.580	Plasma membrane	2	9
**TcZIP6**	Thecc.06G138900.1	1185	394	42.379	9.02	0.479	Plasma membrane	6	9
**TcZIP7**	Thecc.07G016100.1	1077	358	38.431	6.03	0.532	Plasma membrane	7	8
**TcZIP8**	Thecc.07G188800.1	1092	363	38.522	6.45	0.510	Plasma membrane	7	8
**TcZIP9**	Thecc.09G054100.1	1308	435	46.305	6.19	0.256	Chloroplast	9	6
**TcZIP10**	Thecc.09G317600.1	984	327	35.862	6.58	0.446	Plasma membrane	9	8
**TcZIP11**	Thecc.09G352700.1	966	321	34.140	7.13	0.696	Chloroplast.	9	8

kDa - kilodaltons (unified atomic mass unit), pI - isoelectric point and GRAVY – grand average hydropathy.

To examine the diversity of TcZIP gene structure, we compared the exon/intron organizations in the coding sequences of individual genes in cacao. TcZIP genes have several characteristics in terms of gene structure, exhibiting various exon/intron regions (**Figure 1B**). Most TcZIP genes have more than one intron, and the number of introns varied between 1-3 between genes. TcZIP9 has the highest number of introns and TcZIP11 has the lowest number. These results are consistent with those of phylogenetic analysis, in which genes that clustered into the same cluster exhibit similar exon/intron structures. Then we looked for conserved motifs to examine the diversity of motif composition among TcZIPs. As shown in **Figure 1C**, 10 distinct conserved motifs were identified in ZIP transport proteins in cacao. All TcZIPs retained motifs 2, 5, 7 and 8, besides, we found that motifs 2 and 8 are present in the N-terminal region and motifs 5 and 7 are present in the C-terminal region. Subgroup I proteins have high divergence in motifs compared to the other subgroups and two proteins in this subgroup contain the fewest identified domains. All subgroup III and IV proteins have the same motifs 2, 3, 4, 5, 6, 7, 8, 9, and 10. However, motif 9 was not found in two proteins from subgroup I and one protein from subgroup II.

### 3.2 Phylogenetic analysis of TcZIP proteins

**Figure 2:**
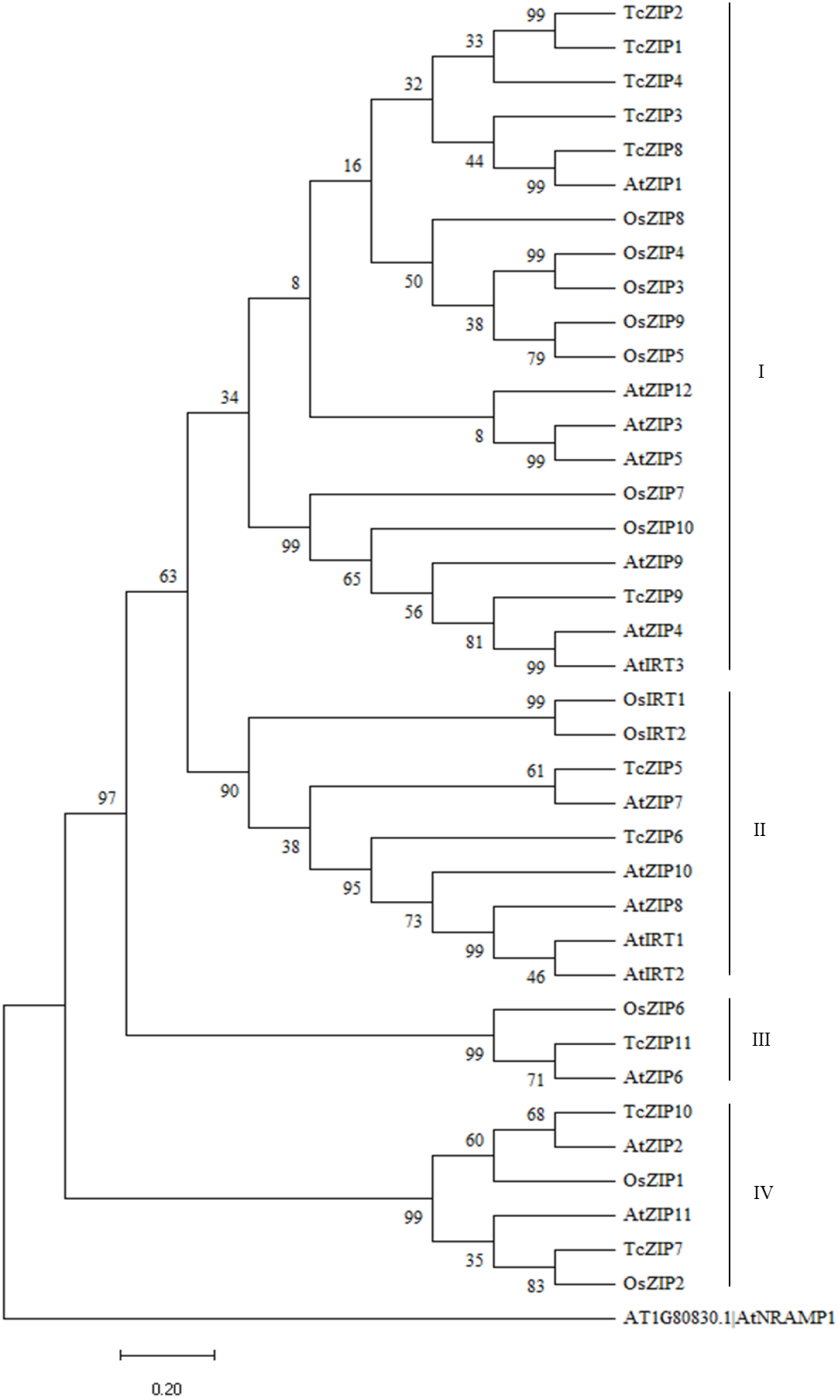
A phylogenetic tree of proteins similar to ZIP from *A. thaliana*, *O. sativa* and *T. cacao*, built using MEGA11 software by the NJ method, with 1000 bootstrap replicates. The beginning of each gene ID contains the code for the species as Tc, *T. cacao*; At, *A. thaliana* and Os, *O. sativa*. The groups are distinguished by different colors.

In the present study, phylogenetic analysis of ZIP transport proteins contained in *A. thaliana*, *Oriza sativa* and *T. cacao* (**Supplementary Table 1**) revealed that AtZIPs, OsZIPs and TcZIPs were divided into four groups. For several protein sequences of the model species used, putative orthologous AtZIPs were found in cacao. Almost all OsZIPs were identified as closely related groups, except for one OsZIP where it had putative orthology with only one TcZIP. Group I with 20 ZIP proteins was identified as the largest clade and group III with three proteins was the smallest clade (**Figure 2**). In group I, only TcZIP1 and TcZIP2 were shown to be closely related. TcZIP8 showed orthology with AtZIP1, which by similarity is closer to TcZIP3, which was not assigned a closer ortholog. The cluster with AtIRT3, AtZIP4, AtZIP9, OsZIP7 and OsZIP10 contained only one cacao ortholog (TcZIP9). In group II AtZIP7, that showed orthology with TcZIP5 and TcZIP6, was marked by being the most distant ZIP member presenting the greatest genetic divergence in contrast to the other TcZIPs. The cluster with AtZIP8, AtZIP10, AtIRT1 and AtIRT2 were the closest model ZIPs. In group III, AtZIP6 showed orthology with TcZIP11. AtZIP2, belonging to the group IV, showed orthology with TcZIP10. In this group, a ZIP member of the *O. sativa* monocot species (OsZIP2) showed orthology with TcZIP7. In general, phylogenetic analysis showed that ZIP transport proteins in cacao were more like *A. thaliana* species than *O. sativa*. Normally, genes grouped in the same group of a phylogenetic tree often reflect that they have similar functional characteristics.

### 3.3 Gene duplication analysis and chromosomal location

**Figure 3:**
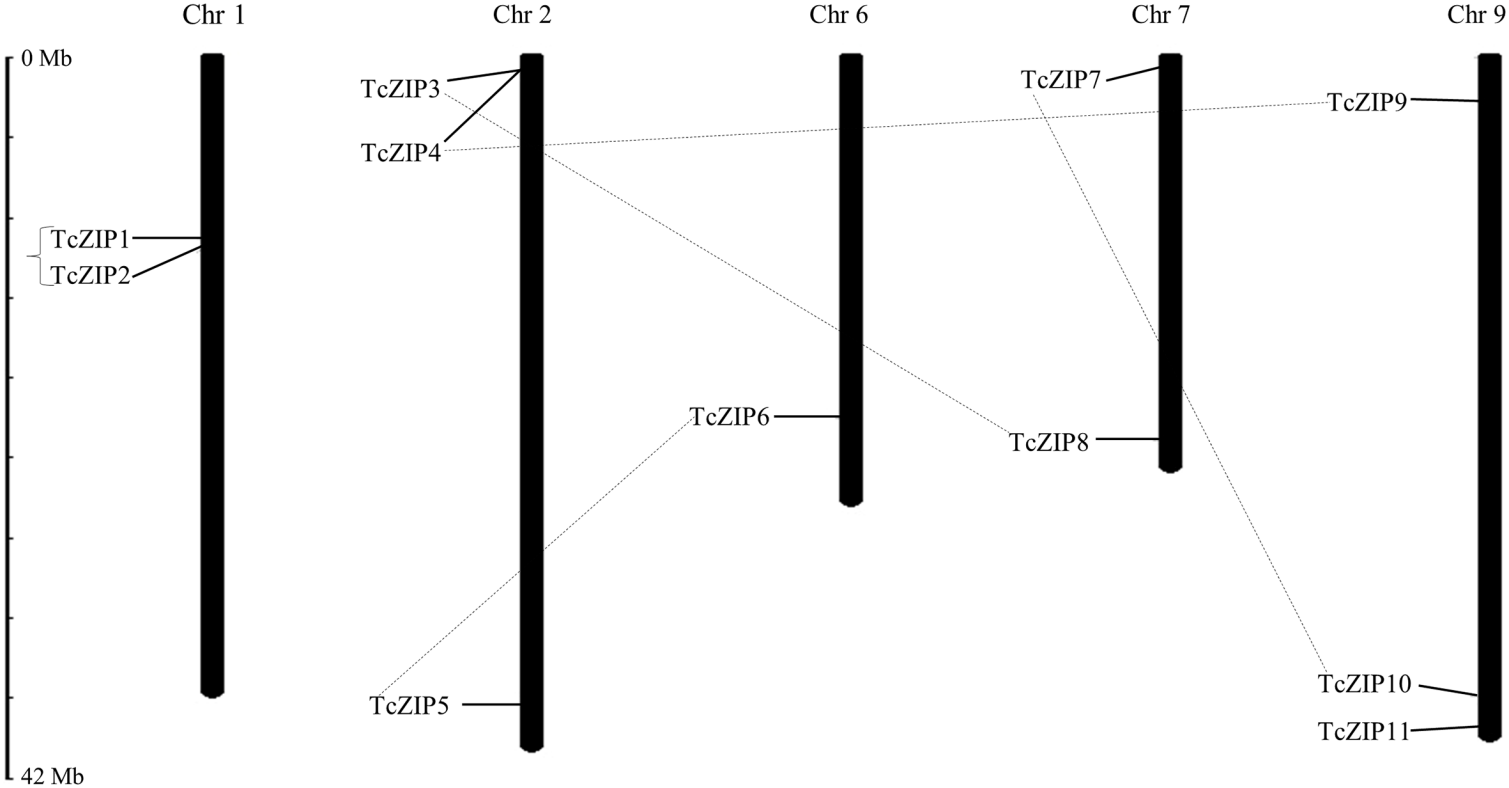
Chromosomal locations and TcZIP genes in cacao. The chromosomal positions of the TcZIP genes were mapped according to information available in Phytozome database. Chromosome numbers are indicated at the top of each chromosome. Genes with segmental duplication are represented by connected dashed lines and tandem duplication are marked by black square brackets. The scale is in megabase (Mb).

Analysis of gene duplications events, particularly tandem and segmental duplications, are important processes implicated in gene family expansion. To detect duplications in the ZIP gene family in cacao, we used the Ka/Ks calculation software. The analysis identified five pairs of paralogous genes in the ZIP family. Based on the duplication analysis, only the TcZIP1 and TcZIP2 genes might be considered as tandem duplicated genes due to their physical proximity. The other four gene pairs showed segmental duplication (**Table 2**). Consecutively, selective constraint analyzes were performed on the five duplicated paralog pairs, from which were calculated the value of synonymous mutations (Ks) and nonsynonymous mutations (Ka). The Ks of the duplicated genes ranged from 0.078 to 0.480. The values of the Ka/Ks ratio, widely used to measure the rate of genetic evolution, also considered as genetic pressure selection, ranged from 0.21 to 0.42 (**Table 2**). The results of this analysis indicated that purifying selection is the main evolutionary force in the ZIP paralog pairs in cacao. It is estimated that the duplication event for the tandem duplicated pairs occurred 5.94 million years ago (Mya) and for the segmentally duplicated pairs occurred 27.26-36.58 Mya. We also performed the physical distribution of the ZIP family members in cacao, using the chromosomal positions of the ZIP genes obtained from the cacao genome in the Phytozome database. We then designated these 11 TcZIP genes according to their physical locations (from the top to the bottom) on the chromosomes. The TcZIP genes were unevenly distributed in five of ten cacao chromosomes. The highest number of duplicated gene pairs was observed on chromosome 2 containing all duplicated genes (**Figure 3**). Chromosome 1 has two genes, chromosome 2 has three members, chromosome 6 has only one, and chromosomes 7 and 9 have 2 and 3 genes, respectively. In general, all five cacao chromosomes contain duplicated genes. Therefore, we hypothesized that the ZIP gene family in cacao is a slowly evolving gene family during the evolution process.

**Table 2:** Ka/Ks analysis and estimated divergence time for ZIP genes pairs in cacao.

Paralogou pair	Ka	Ks	Ka/Ks	Duplication date (MYA)	Duplicate type
**TcZIP2-TcZIP1**	0,027	0,078	0,345	5,94	Tandem
**TcZIP3-TcZIP8**	0,097	0,452	0,214	34,42	Segmental
**TcZIP4-TcZIP9**	0,191	0,455	0,420	34,65	Segmental
**TcZIP10-TcZIP7**	0,143	0,480	0,298	36,58	Segmental
**TcZIP5-TcZIP6**	0,124	0,358	0,345	27,28	Segmental

### 3.4 Promotor region analysis

**Figure 4:**
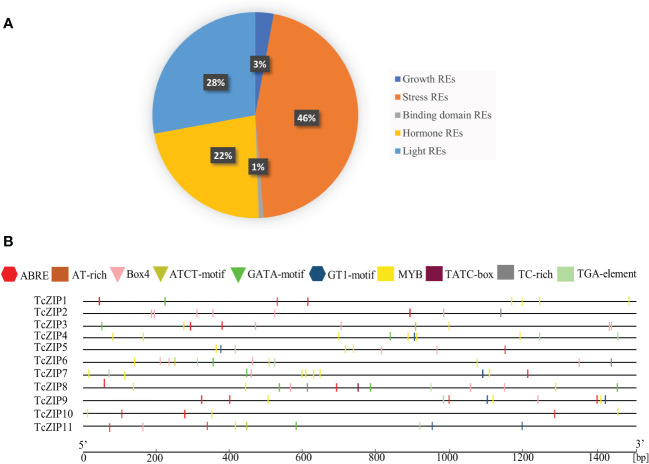
Analysis of TcZIP promoter region. **(A)** Cis-regulatory elements in TcZIP genes promoter regions. Represents the extent of various types of regulatory elements based on function, such as light, hormone, growth, stress and binding domain, excluding the TATA box, CAAT box and **(B)** Location of cis-regulatory elements in the 1.5 kb 5’ upstream region of each TcZIP gene involved in the stress response.

To understand the regulatory mechanisms of TcZIP gene transcription, we used the sequences of all TcZIP genes to predict the cis-acting regulatory elements. We identified and analyzed for the first time cis-regulatory putative elements in promoter regions of ZIP genes in cacao (**Supplementary Table 2**). The cis-regulatory elements were divided it into five categories, considering their function. The presence of a lot of transcription factors was associated to stress response in general (46%), light responsive element (28%), hormone responsive elements (22%), growth response elements (3%) and binding sites related to DNA and proteins (1%) (**Figure 4A**). In this study, we focus on cis-regulatory elements in stress response. For example, ABRE is involved in abscisic acid responsiveness; the TGA-element is involved in auxin response; Box4, GATA-motif, GT1-motif, and ATCT-motif are involved in root responsiveness. Myoblastosis and Tc-rich are involved in defense and stress response. ABRE is present in most genes, except TcZIP4 and TcZIP6. GATA-motif is present in greater quantity in TcZIP8. MYB was identified as the most abundant element in TcZIP genes, except for TcZIP2. TATC-Box, TC-rich and ATCT-motifs were the elements with the lowest abundance, being present in a few genes. In addition, cis-regulatory elements related to transcription modulation, endosperm and meristem expression, leaf differentiation and development, and circadian control were found in lower abundance in the promoter region. Also new cis-regulatory elements were found, these elements were categorized as putative elements without analysis, due to the little information we have about them. Full details of all identified elements except TATA-box and CAAT-box along with sequence and function are given in **Supplementary Table 1**. In this way, TcZIP genes can be regulated by various environmental factors and developmental changes. We also identified that the duplicated gene pairs do not share the same element distributions, these observations indicate that evolutionary changes resulting from duplication events conferred neofunctionalization on TcZIP genes during their divergence and evolution (**Figure 4B**).

### 3.5 Analysis of post-translational modifications and 3D structure of TcZIP proteins

**Figure 5:**
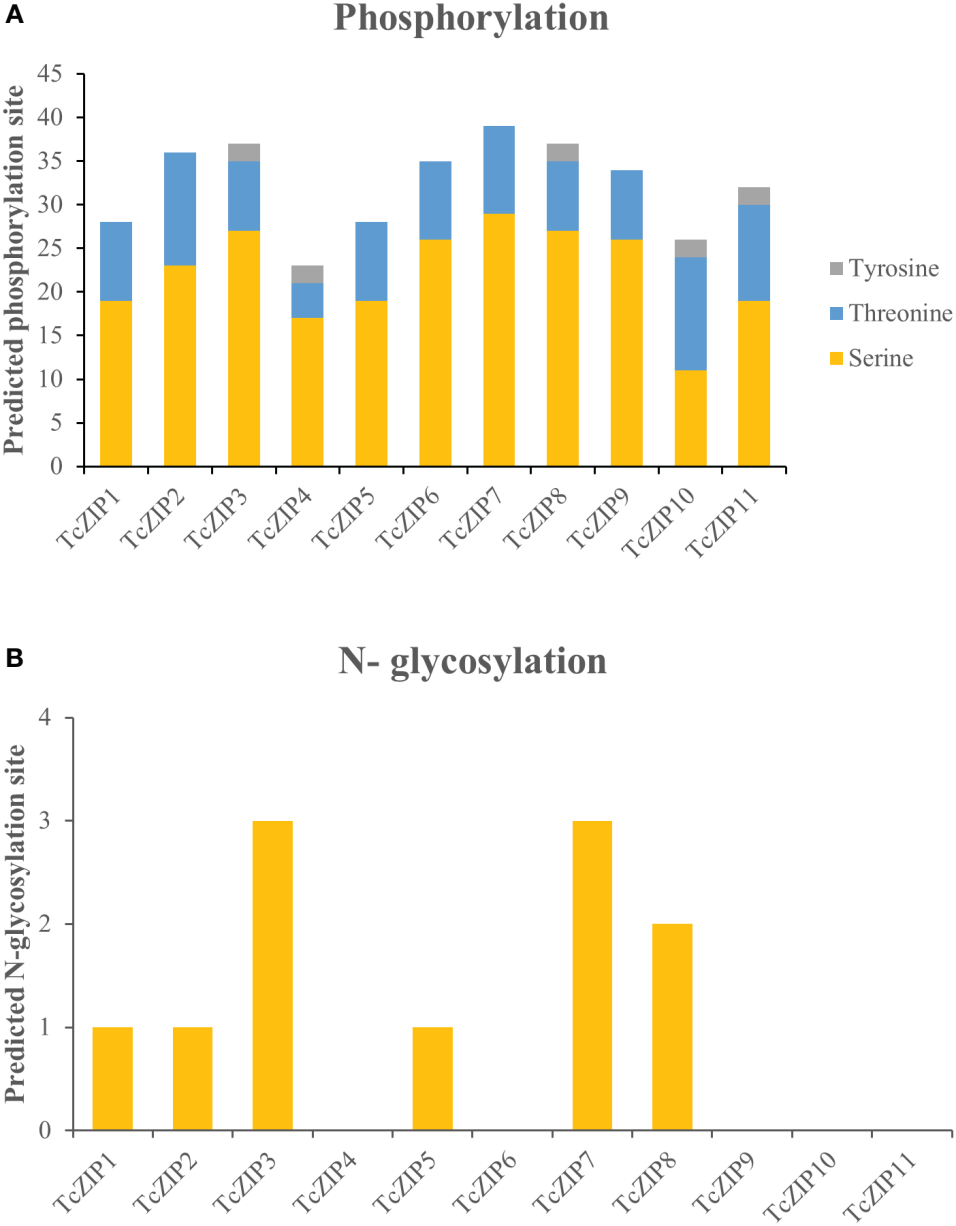
Prediction of post-translational modifications in amino acid sequences of the Zn/Fe-regulated transporter genes in *T. cacao* (TcZIP), based on the **(A)** Predicted phosphorylation site and **(B)** N-glycosylation site.

**Figure 6:**
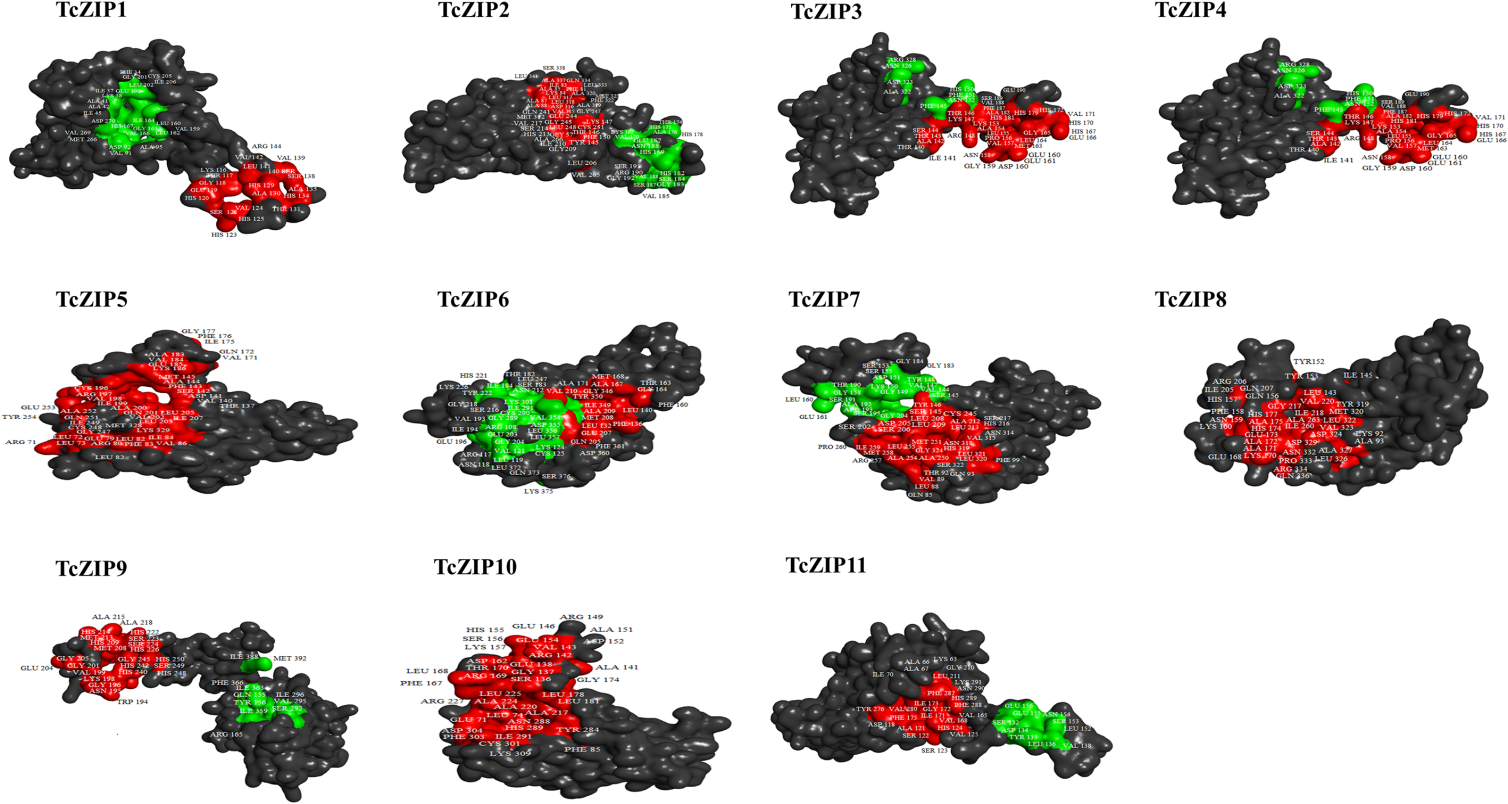
Identification of activity sites in the predicted 3D structure of all ZIP transport proteins studied. The distribution of major pocket protein sites in the ligand regions is highlighted in the three-dimensional structure with its main residues.

In the present study, post-translational modifications of TcZIPs were predicted in terms of phosphorylation and N-glycosylation (**Figure 5**, **Supplementary Table 3**). We identified a total of 355 potential phosphorylation events in serine, threonine and tyrosine amino acids belonging to TcZIP proteins. The predicted potential phosphorylation events were related to serine (243) followed by threonine (102) and then tyrosine (10) (**Figure 5A**). Among the TcZIPs proteins, most of the phosphorylation sites (39 sites), were predicted in TcZIP7 and followed by five proteins that ranged from 34 to 37 sites. Furthermore, two TcZIPs proteins, including TcZIP4 and TcZIP10 were the ones with the lowest predicted phosphorylation sites (**Figure 5A**). The potential sites of N-glycosylation of the ZIP transport proteins were also predicted, except for six proteins TcZIP4, TcZIP6, TcZIP9, TcZIP10 and TcZIP11. In addition, several proteins showed potential N-glycosylation with few sites (**Figure 5B**). The N-glycosylation results indicate that TcZIP3 and TcZIP7 are the proteins with the most glycosylation sites (3 sites each), while only two glycosylation sites were predicted in TcZIP8. Next, TcZIP1, TcZIP2 and TcZIP5 were observed with a glycosylation site (**Table S1**). The three-dimensional structures of all candidate proteins were modeled with >90% confidence, as well as the percentage of residues in the most energetically favorable region are shown in the Ramachandran plot (**Supplementary Figure 1**). The predicted 3D structures showed the presence of α helices, turn, random coils, bend and β sheets. The α helices, turn, random coils, bend were the most abundant, on the contrary, the occurrence of β sheets were scarce in all TcZIP proteins, appearing in only two proteins, TcZIP7 and TcZIP10 (**Figure 6**, **Supplementary Figure 2**). The diversity in predicted structures may be due to their different ion transport in response to multiple environmental stimuli. Besides, the active sites of proteins, surface pockets and cavities were predicted according on structure. Based on the results, different surface pockets were observed in the TcZIP proteins and the main amino acids involved in the metal binding sites as well as in the function of the TcZIP proteins were predicted (**Figure 6**). Leucine (LEU), alanine (ALA), glycine (GLY), serine (SER), lysine (LYS) and histidine (HIS) residues were the most predicted, being related to the surface pockets of almost all putative ZIP transport proteins (**Supplementary Figure 3**). Overall, we infer that these amino acids are recognized as the key residue at pocket sites, allowing for metal binding in TcZIP transport proteins. In addition, the ZIP proteins in cacao showed differences in the distribution of pocket sites that directly affect their functions. These results suggest the importance of these residues in the pocket sites, as well as the distribution of surface pockets and cavities, which may influence metal affinity and, ultimately, cellular functional performance.

### 3.6 Systems biology

**Figure 7:**
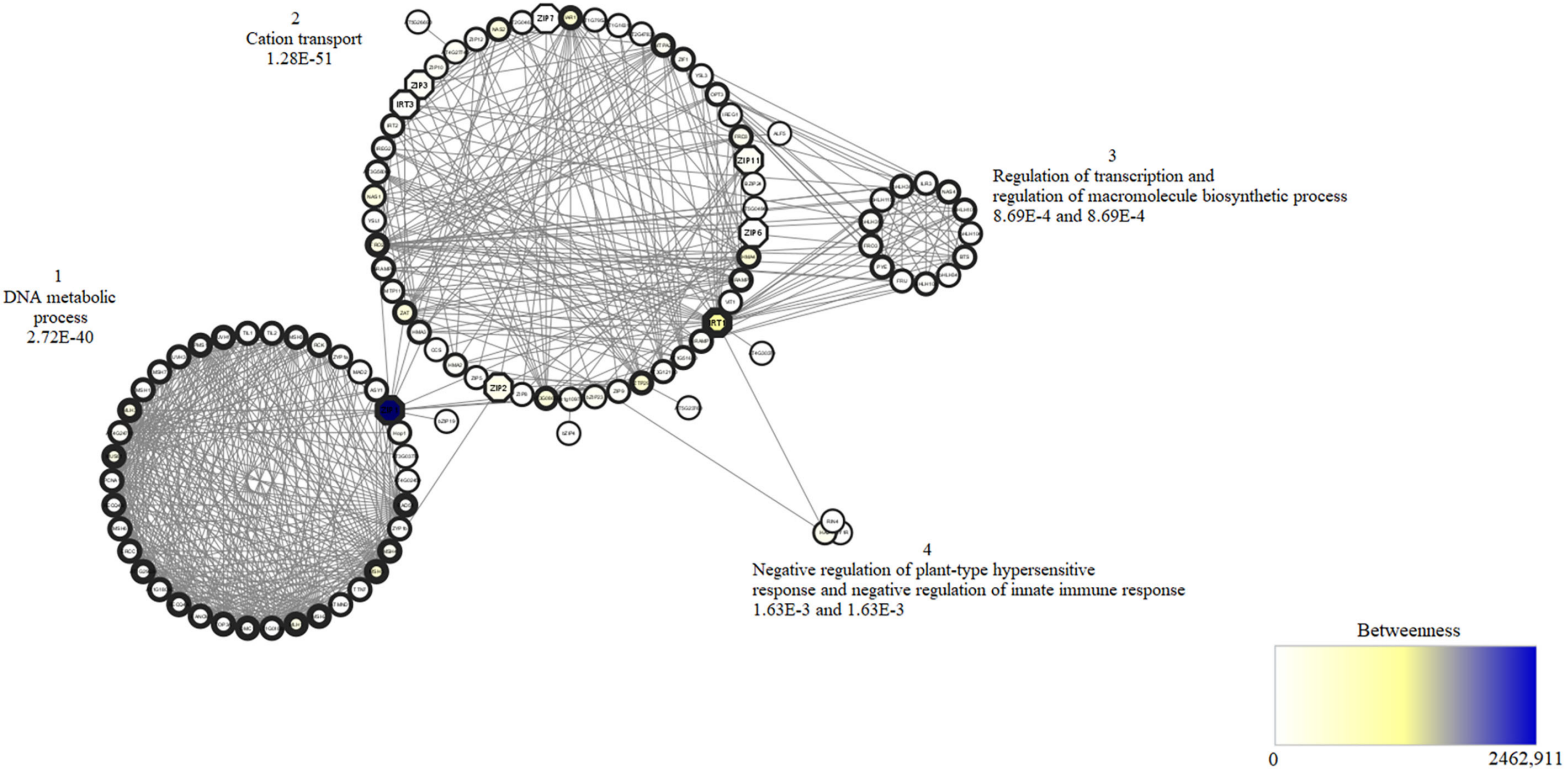
View of an interaction network of proteins from *A. thaliana* homologous to those identified in *T. cacao*. The betweenness value is represented by the color of the nodes; the lightest is the lowest value and the darkest is the highest value. This represents the node’s ability to join multiple clusters or groups of nodes. The edge width of nodes represents the node degree value, so the larger the edge width, the larger the value and vice-versa. The node degree property represents the number of connections that cross a single node. Clusters are subsets of nodes that are primarily connected to each other. For each cluster, a biological process with the lowest corrected value was assigned according to BiNGO tool.

The interaction network of proteins containing graphical representations of sets of nodes connected by edges was constructed from *A. thaliana* proteins, identified homologs of the transport family in *T. cacao* (**Table 1**). In this study, the interactome, consisting of 109 nodes (representing the individual proteins) was interconnected by 706 edges (each one representing an interaction). When all interactome proteins were analyzed four clusters were evidenced (**Figure 7**). The protein interaction network contains 22 proteins qualified as bottleneck proteins (betweenness value above average) important to connect several clusters that, in this case, represented the biological processes, among which five are homologous to those identified in *T. cacao*. The network also contains 53 highly connected proteins, qualified as hubs proteins (above average node degree value), of which two proteins are homologous to the proteins identified in *T. cacao*. Moreover, two of the homologous proteins identified have both characteristics in the network (bottleneck and hub). Regarding biological processes, cluster 1 is made up of 39 proteins, associated as they are involved in the DNA metabolic process, such as MutS Homolog (MSH). In this cluster, a homolog corresponding to ZIP1 was found, which has both characteristics. On the other hand, cluster 2 contains the largest number of proteins in the network. This cluster is involved in cation transport-related proteins. In turn, seven homologous proteins identified in *T. cacao* corresponded to proteins ZIP2, ZIP3, ZIP6, ZIP7, IRT1 and IRT3, respectively, these proteins have the characteristic of bottleneck proteins. It was shown that the homologous ZIP bottleneck proteins interact with other transport proteins such as heavy metal P-type ATPase (HMA3 and HMA4), yellow stripe-like (YSL3), auxin conjugate–resistant (IAR1), natural resistance-associated macrophage protein (NRAMP2) and metal transporter CDF (MTPA2). Clusters 3 and 4 have the lowest amount of related proteins with regulation of transcription, regulation of macromolecule biosynthetic process, negative regulation of plant-type hypersensitive response and negative regulation of innate immune response. Of the 11 putative proteins identified in *T. cacao*, using the STRING database with the parameters described, only 8 of the 15 ZIP proteins from *A. thaliana* (including the IRT) showed high homology. These 8 are represented in the form of octagons on the network.

## 4 Discussion

The ZIP family is a group of metal ion transporter genes found in various kingdoms including plants (Guerinot, 2000). This family is involved in the transport of various essential and non-essential metal ions. In the present paper, we performed a general analysis of the ZIP gene family in cacao, including analysis of its phylogeny, chromosomal location, gene structure, conserved motifs, prediction of three-dimensional structure and protein-protein interactions (PPIs). A total of 11 ZIP genes were identified in the cacao genome (**Table 1**), which is fewer than the ZIP members identified in the model species *A. thaliana* (15) and a similar number as in *Solanum tuberosum* (12) (Li et al., 2020). Eventually the ZIP family is relatively small, so far the smallest number of ZIP members has been found in *Zea mays* (Li et al., 2013). Sequence analysis showed that the TcZIP proteins bear similarity to the previously studied ZIP families (Guerinot, 2000). Specifically, TcZIP members encode proteins with lengths from 309 to 435 aa, in agreement with the predicted range in known plants (Guerinot, 2000). Studies in perennial plants such as *Poncirus trifoliata* reveal that ZIP members encode proteins with 334 to 419 aa (Fu et al., 2017). Most of the TcZIPs were predicted to be located in the plasma membrane, similarly to AtIRT1, OsZIP4, OsZIP5, HvIRT1 and VvZIP3 (Vert et al., 2002; Ishimaru et al., 2005; Pedas et al., 2008; Lee et al., 2010; Gainza-Cortés et al., 2012; Krausko et al., 2021), and also predicted in the ZIPs members of the *Poncirus trifoliata* and *Zea mays* genomes (Li et al., 2013; Fu et al., 2017). However, two TcZIP genes are predicted to be located in the chloroplast. Certainly, ZIP genes can be expressed in various parts of plants, and they can be located in the membrane of internal organelles such as chloroplasts (Ajeesh Krishna et al., 2020). Furthermore, between 6-9 MTs were identified in TcZIP proteins, similarly to ZIPs *Poncirus trifoliata* and *Zea mays* (Li et al., 2013; Fu et al., 2017).

On the phylogeny analysis, the 11 TcZIP genes identified are grouped into four subgroups, similar to ZIP genes of *A. thaliana* (Mäser et al., 2001). In each group, most genes have characteristics of the exon/intron structure and relatively conserved motif compositions in recent paralogs, except for the exon/intron structures of two paralog pairs, in which some differences still exist (**Figure 1**). For instance, structural analysis showed that TcZIP4 and TcZIP9 have different numbers of introns, in contrast to HD-zip gene family in *Glycine max* that showed relatively conserved exon/intron structure among the paralog pairs. This is due to the few insertions and deletions that accumulated along the evolution (Schmutz et al., 2010). Studying the structure of the exon-intron gene may provide important clues to the evolution of the gene (Freeling, 2009), because genes in the same group can have similar functions. The interspecies phylogeny results show that they are grouped into four distinct groups, in which the first group has the highest number of proteins. Although the groups identified are different from those found by (Li et al., 2020), our results showed consistency in the groups identified in the phylogenetic relationships made in the *Vitis vinifera* perennial species (Gainza-Cortés et al., 2012). Thus, the discrepancy with that presented by Li et al. (2020) may be due to the numbers of genomes used; in this one ZIP members of five plant genomes were used.

The phylogenetic study showed putative orthologous ZIP genes, unexpectedly there was also an orthologous pair of monocots and dicots (TcZIP7-OsZIP2), suggesting that the orthologous pair originated from common ancestral genes that existed before the divergence of monocots and dicots. Due to the existence of several paralogous genes in the TcZIP family, it is possible to confirm that the cacao tree underwent two duplication events after the monocotyledonous/dicotyledonous split. This suggests that most of the ZIP genes in cacao expanded in a species-specific manner. Gene duplication plays an important role in the evolution of the organism; the generation of new genes will allow organisms to adapt to different environments. Two main evolutionary mechanisms have been attributed to gene duplication, including segmental and tandem duplication (Horan et al., 2005; Kong et al., 2007). In our analysis, we found five pairs of duplicated ZIP genes, the distribution was preferentially for segmental duplications, suggesting that segmental duplication was the main driver for the expansion of the ZIP gene family in cacao. Certainly segmental duplication is the mechanism frequently discovered in plants, as most plants are diploidized polyploid and retain numerous duplicated chromosomal blocks within their genomes (Cannon et al., 2004; Del Pozo and Ramirez-Parra, 2015). Estimates of the Ka/Ks ratio were utilized to measure the selection rate of genetic pressure. Based on these results, we propose that purifying selection is the main evolutionary force in the ZIP paralog pairs in cacao. Since, based on the theory of natural selection, when the ratio Ka/Ks >1 indicates positive selection, while the Ka/Ks =1 indicates neutral selection, and finally when the ratio Ka/Ks <1 indicates negative or purifying selection (Gaut et al., 1996). Previous studies indicate that the cacao genome has undergone lineage-specific scrambling events from the paleohexaploid ancestor. It is estimated to have occurred 123 million years ago (MYA), and at least 11 chromosomal fusions have occurred to achieve the recent structure of ten chromosomes (n), estimated to have occurred around 59 MYA (Argout et al., 2011). By calculating the duplication dates of the paralog pairs, we showed that all segmental duplication events in the ZIP family in cacao occurred after the evolutionary scenario that gave rise to the ten chromosomes. The results indicated that the gene pair with tandem duplication appeared 5.99 MYA ago, being the most recent event. Almost all cacao chromosomes contain duplicated genes, so we assume that the ZIP gene family is a slowly evolving gene family, in the same way as was found in the TCP gene family in *Z. mays* (Chai et al., 2017). Evidently segmental duplication played an important role in the expansion of ZIP genes. Furthermore, the cis-acting elements that control many biological processes and responses to different stimuli through a regulatory system allow genes to have important unique combinations (Ibraheem et al., 2010). In this study ABRE, TGA-element, Box4, GATA-motif, GT1-motif and ATCT-motif, Myoblastosis and Tc-rich are essential elements for the stress response. These cis-acting elements are abundant in TcZIP as well as in potato ZIP members (Li et al., 2020).

Predicting the 3D structure and identifying the pocket site of proteins provides the structural and microenvironment basis for proteins to perform functions such as ligand binding, enzymatic activity and DNA interaction (Edelsbrunner et al., 1998; Tian et al., 2018). Analysis of the three-dimensional structure showed that α-helices conformations are predominant and β-sheets are practically absent, in the same way as reported in ZIP protein structures in other species (Ajeesh Krishna et al., 2020), showing that α-helices play an important role in ZIP proteins. All predicted TcZIP models have structural similarity to the recent Zrt-/Irt-like protein (ZIP) crystal structure of *Bordetella bronchiseptica* (Zhang et al., 2020), which has a key function in the transport of bivalent transition metal ions (Zn, Fe, Cd, Mn, Ni, Co e Cu) (Antala and Dempski, 2012; Jeong and Eide, 2013). Pocket sites and predicted cavities were diverse in the structures of TcZIP proteins, which provides valuable insights into protein function based on metal-binding sites. The amino acid residues LEU, ALA, VAL, GLY, SER, LYS and HIS were frequently predicted within the pockets for almost all candidate proteins. Among the residues found, GLU and HIS were identified in previous studies as part of the substrate binding sites (Antala et al., 2015). Another study showed that the residues GLU, HIS, ASP and CYS coordinate iron binding in various wheat proteins (Verma et al., 2017). A later study shows that LYS, PHE and SER residues also contribute to the transport process (Gyimesi et al., 2019). These residues were found in pocket sites of TcZIP proteins, suggesting they are important for transport activity. Post-translational modifications are modifications in the side chain of amino acids in some proteins after their biosynthesis, these modifications regulate several cellular processes (Ramazi and Zahiri, 2021). All ZIP members in cacao have potential phosphorylation sites. Previous studies have revealed that ZIP proteins undergo many phosphorylations. Protein phosphorylation by a kinase (CIPK23) and IDF1-mediated polyubiquitination are important for efficient endosomal sorting and vacuolar degradation of IRT1. Moreover, phosphorylation as a subsequent process of protein-metal binding helps to optimize the process of uptake and protection of plants from harmful metals (Dubeaux et al., 2018). It is also known that phosphorylation at SER residues of ZIP7 in humans dramatically increases its transport activity (Hu, 2021). In addition, potential sites for N-glycosylation were also predicted. Six TcZIP proteins are glycosylated at the N-terminal. In the current study, TcZIP3 and TcZIP7 are the highlighted proteins (three sites both). This indicates that ZIP proteins in cacao have few sites for N-glycosylation. It has been demonstrated in previous studies, proteins from the ZIP gene family are rarely glycosylated, and those that show N-glycosylation exhibit values of less than four sites as observed in ZIP4, ZIP6, ZIP8 and ZIP14 (Taylor et al., 2003; Wang et al., 2004; He et al., 2006; Girijashanker et al., 2008).

Studies of PPIs have revealed valuable information about the possible interactions and processes involved in target proteins. The function of a protein is governed by its interaction with other proteins within a cell, PPIs establish one of the most decisive conditions for sustaining life in living organisms (Yilmaz et al., 2022). A systematic analysis of the system biology was performed to obtain a broader view of the processes that evolve the ZIP transport proteins homologous to those identified in cacao. In this study, we identified bottlenecks and hubs proteins (Verli, 2014) and 109 proteins constituted the interactome. Proteins qualified as bottlenecks proteins (betweenness value above average) are of importance because they are related to their ability to connect several clusters, which, in this case, represent biological processes. The network also contains 53 highly connected proteins, qualified as hubs proteins (above average node degree), since these proteins have the characteristics of having an important regulatory function within the interactome. The ZIP1 protein with both characteristics (bottleneck and hub) allows the junction between cluster 2 related to cation transport and cluster 1 related to DNA metabolic process. Surprisingly, the interaction of ZIP members reveals other processes besides the transport of cations. Previous studies show that the ZIP1 overexpression leads to apoptosis of tumor prostatic epithelial cells, due to the increase in the concentration of Zn (Samavarchi Tehrani et al., 2019), which leads us to believe that this homeostatic Zn dysregulation causes damage to DNA molecule and cell replication. DNA damage interferes with the development of organisms. Molecular mechanisms for metal toxicity in plants have been described previously (Gill, 2014). A complex network of proteins is activated to protect DNA. The DNA repair response includes different pathways in plants (Tuteja et al., 2001) that are similar to those used in other organisms. For example, MSH family proteins found in cluster 1 can form MSH2/MSH6 and MSH2/MSH7 complexes important in recognizing nucleotide mismatches in *A. thaliana* (Moura et al., 2012), in this way, they mitigate the damage resulting from metal toxicity. Moreover, in cluster 2 with proteins related to the cation transport process, it was shown that the bottleneck proteins ZIP11, ZIP2, ZIP3, and IRT1 interact with other transport proteins.

P-type ATPases (HMAs) are another family of metal transporters found in various kingdom including plants (Baxter et al., 2003). HMA proteins play an important role in Zn, Cd, Pb and Cu homeostasis and have been expressed in various tissues (Hussain et al., 2004; Mills et al., 2003), which demonstrates that they are involved in compartmentalization and detox (Andrés-Colás et al., 2006). Thus, AtHMA3 plays a role in the detoxification of biological (Zn) and non-biological (Cd, Co, and Pb) heavy metals, participating in their vacuolar sequestration, a unique function for a P1B-2 ATPase in a multicellular eukaryote (Morel et al., 2009). The Yellow Stripe-Like (YSL) protein can mediate the transport of metals in the form of a complex. Heterologous expression of BjYSL7 revealed the transport of Fe, Cd and Ni (Wang et al., 2013) using a metal-nicotianamine complex. Similarly SnYSL3 revealed the transport of Fe, Cu, Zn and Cd (Feng et al., 2017). IAR (IAA-Ala–resistant) gene encode a protein involved in auxin metabolism or response. The protein encoded by the IAR gene has several His-rich regions, with structural features shared with the ZIP family of transporters (Lasswell et al., 2000).

Natural resistance-associated macrophage proteins (NRAMP) are a family of metal ion transport proteins identified in yeast, insects, mammals and plants (Cellier et al., 1995) and have substrate specificity for various metal ions, including Cd (Nevo and Nelson, 2006). Recently, several members of NRAMP have been identified in cacao, with NRAMP5 being a potential Cd transporter in cacao (Ullah et al., 2018). Metal transporter CDF proteins encode proton antiporters that effuse heavy metals out of the cytoplasm (Gaither and Eide, 2001). The expression of CDF genes such as MTP1 confers Zn tolerance, as well as knockdown in mtp1 mutants produces Zn-sensitive phenotypes, demonstrating the role of MTP1 in Zn homeostasis (Kobae et al., 2004; Desbrosses-Fonrouge et al., 2005). Studies show that the MTP5 and IAR1 genes transport metals in an antagonistic way, modifying the intracellular ionic composition to influence the activity of the metallohydrolase responsible for hydrolyzing the conjugates with of indole-3-acetic acid (IAA), influencing the homeostasis of IAA (Rampey et al., 2013).

Knowing about the proteins of our interactome, we can infer that there is a complex network of interaction that mediates metals homeostasis in cacao, whose ZIP family is a part of this complex. The activities of import to cell, distribution to organelles and export from the cell, need a controlled and balanced activity between several transporters. Transcriptional control is an important factor in the regulation of cellular homeostasis. Although little is known about the transcriptional profiles in cacao and the main transcription factors that mediate metal homeostasis, this analysis of ZIP transporters PPI contributes to the understanding of a complex network of transport proteins that interact and possibly coordinates from the entry of metallic ions to their use in different tissues and deposition in cacao beans.

## 5 Conclusion

In summary, 11 ZIP genes were identified using the cacao genome for the first time. All sequences analyzed suggest that they have basic characteristics of previously studied members of the ZIP family. Among genes identified, three including TcZIP1, TcZIP2 and TcZP4 are the most distant from their counterparts in the model species. Segmental and tandem duplication have been identified as the main patterns contributing to the expansion of the ZIP gene in cacao. All TcZIP play important roles in the uptake and distribution of transition metals, including the toxic element cadmium, according to pocket site prediction analyses. This study provides essential and comprehensive information for ZIP genes in cacao, but we propose that further work is needed to delve into exact subcellular and tissue localization, gene expression between different genotypes, and KO analysis (gene knockout).

## Data availability statement

The datasets presented in this study can be found in online repositories. The names of the repository/repositories and accession number(s) can be found in the article/**Supplementary Material**.

## Author contributions

DP and A-AA performed the original design preparation, methodology, validated the data, wrote and edited the manuscript and visualized the data. CP and BS performed the formal analyses, curated data and revised the manuscript. A-AA and CP performed resource administration, project administration and financing acquisition and oversaw the data. All authors contributed to the article and approved the submitted version.
