# Supplementary material for: Zinc/iron-regulated transporter-like protein gene family in Theobroma cacao L: Characteristics, evolution, function and 3D structure analysis
Source: Front Plant Sci. 2023 Feb 28;14:1098401. doi: 10.3389/fpls.2023.1098401 (PMC10012423; doi:10.3389/fpls.2023.1098401)
Supplement: Supplementary file 1 [file DataSheet_1.docx]

**TcZIP1 TcZIP2 TcZIP3**


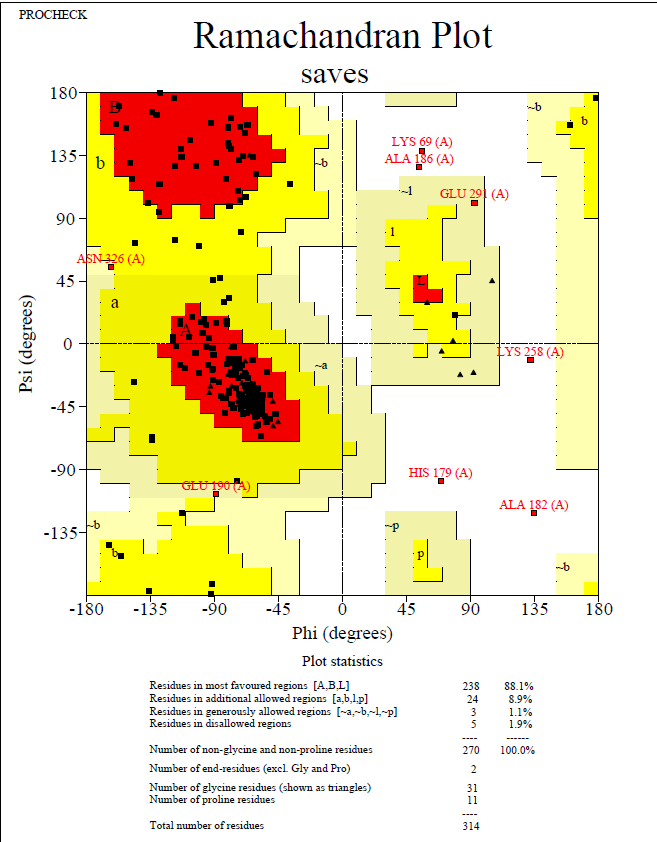

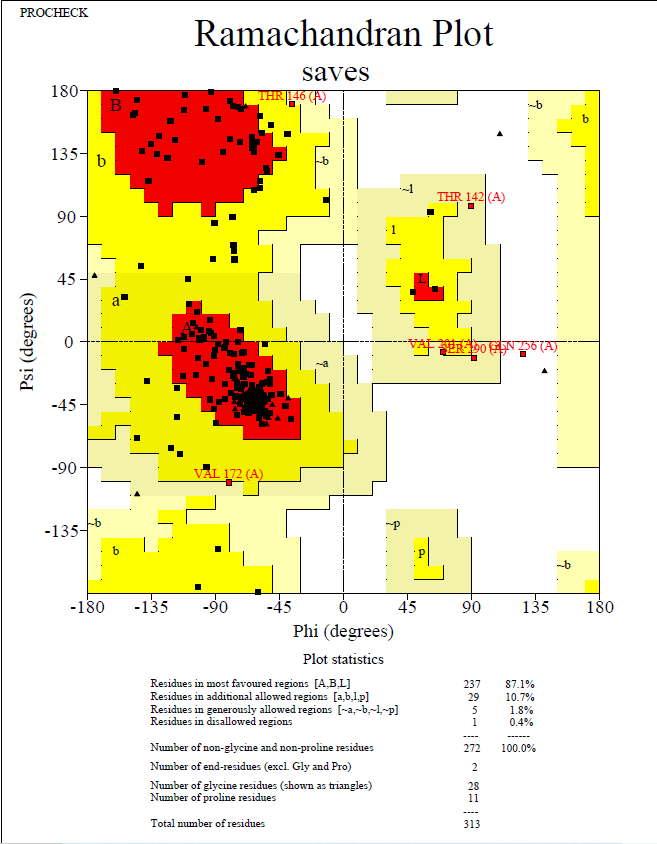

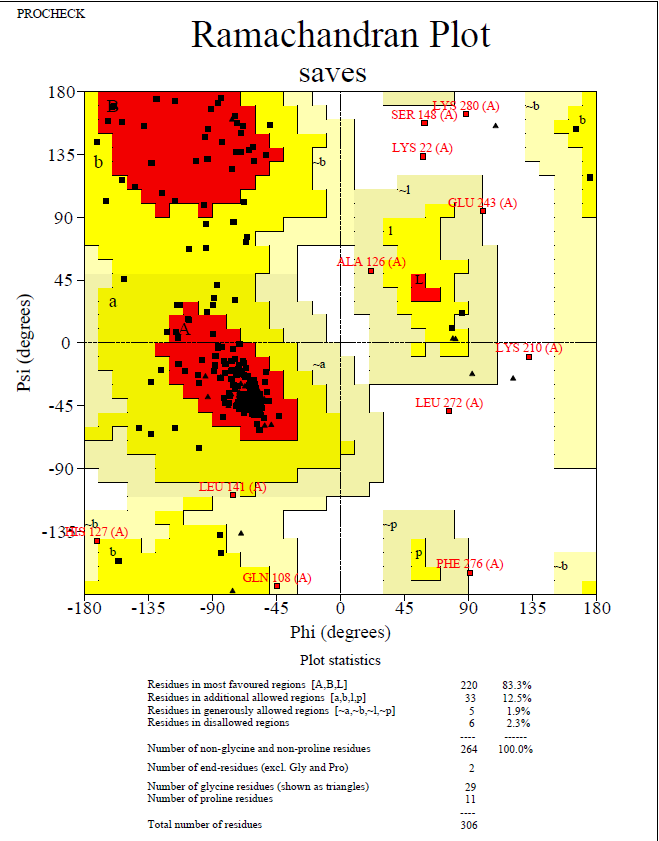


**TcZIP4 TcZIP5 TcZIP6**


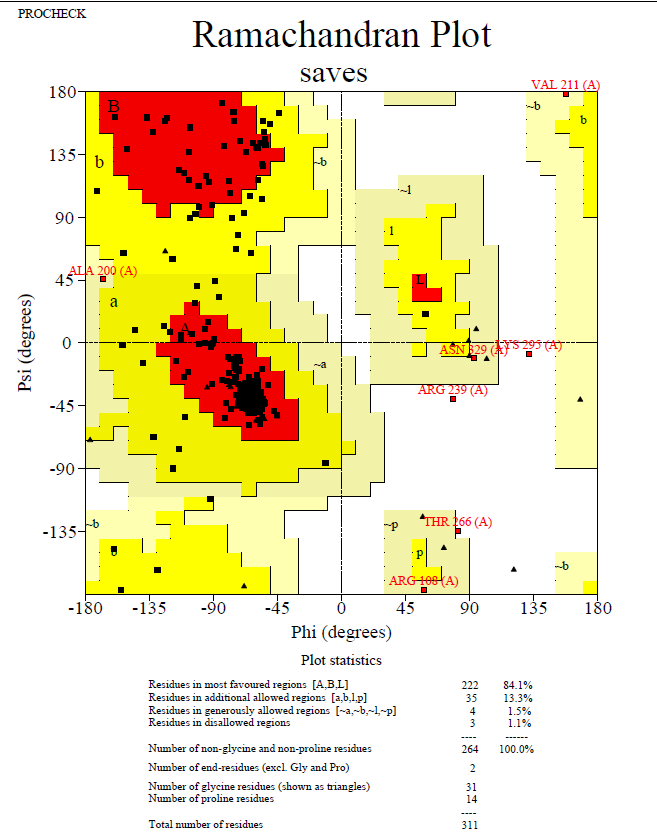

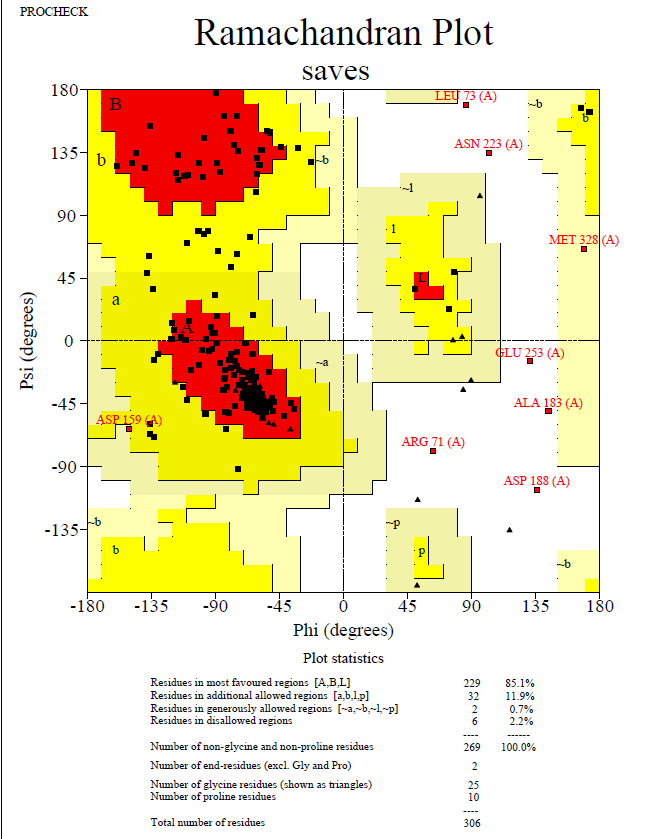

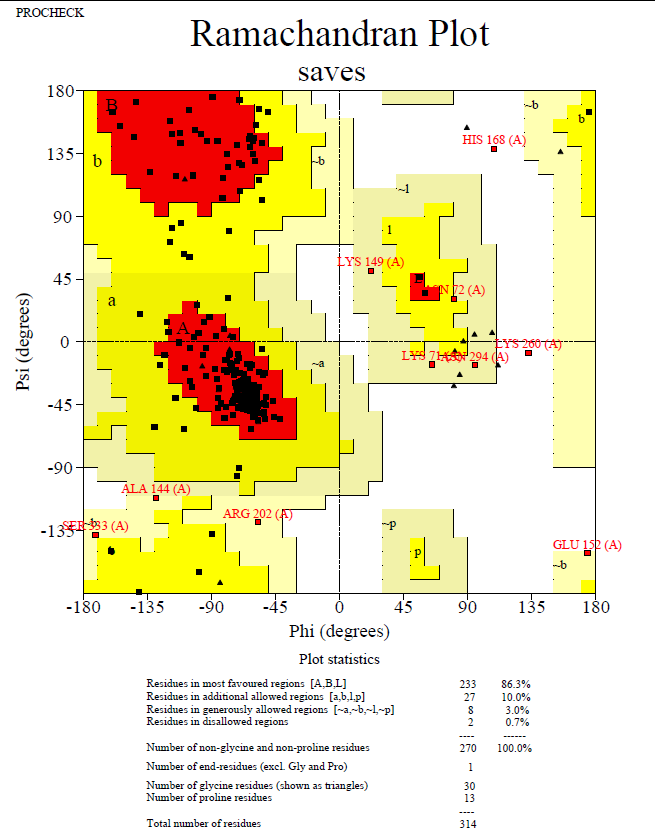


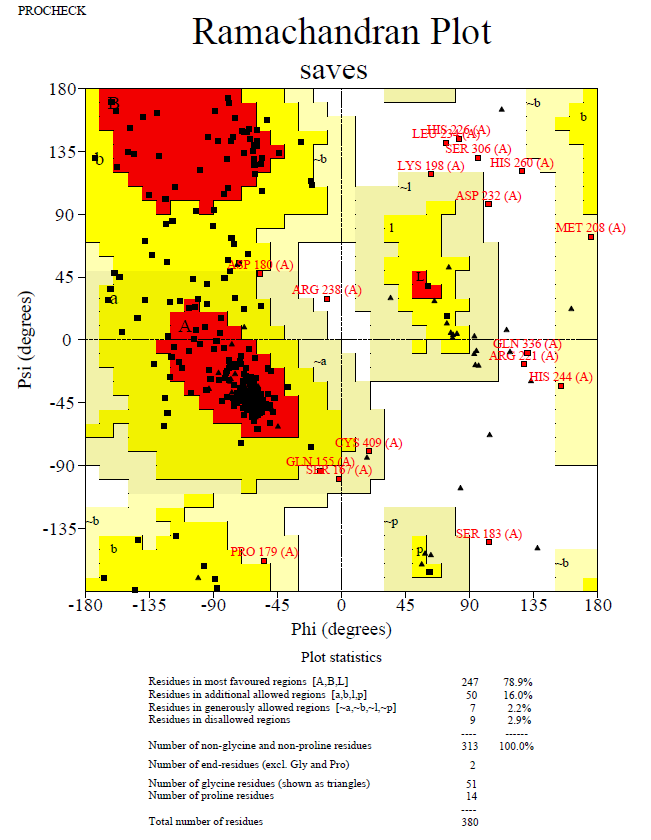

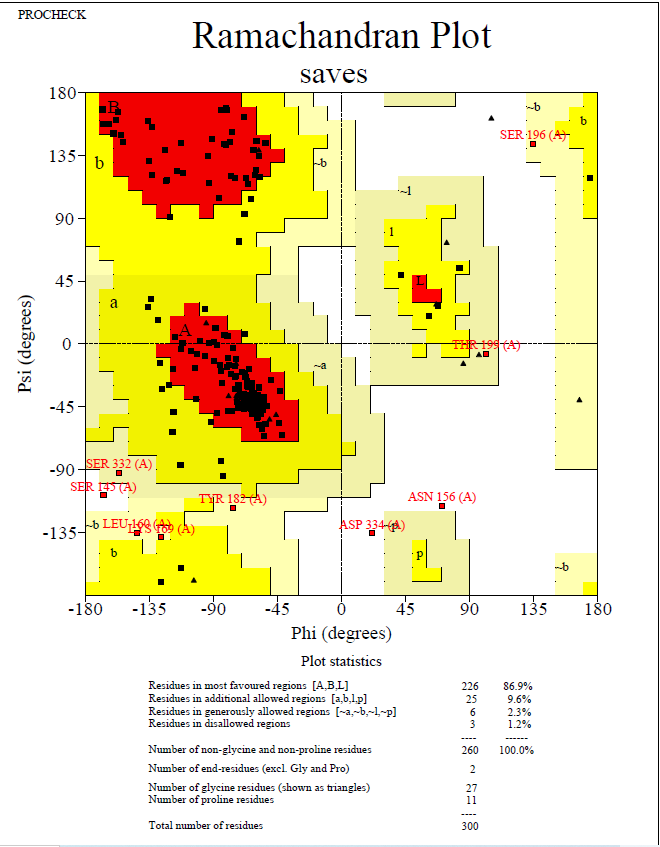
 **TcZIP7 TcZIP8 TcZIP9**


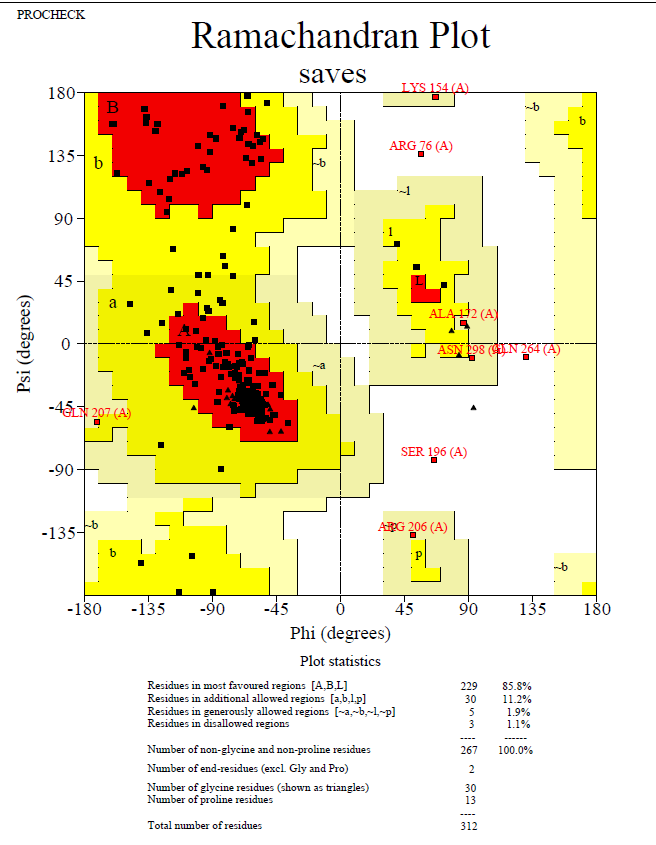


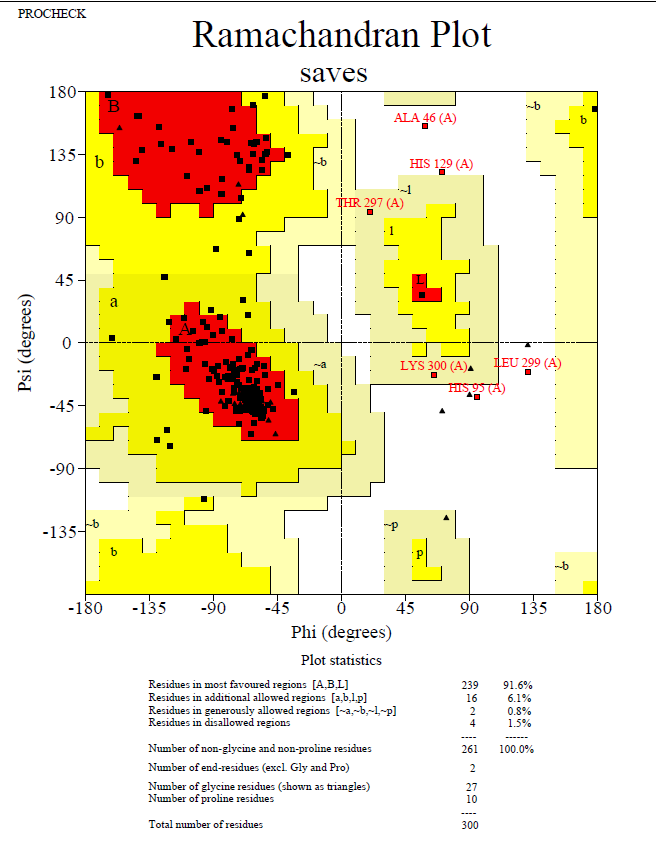

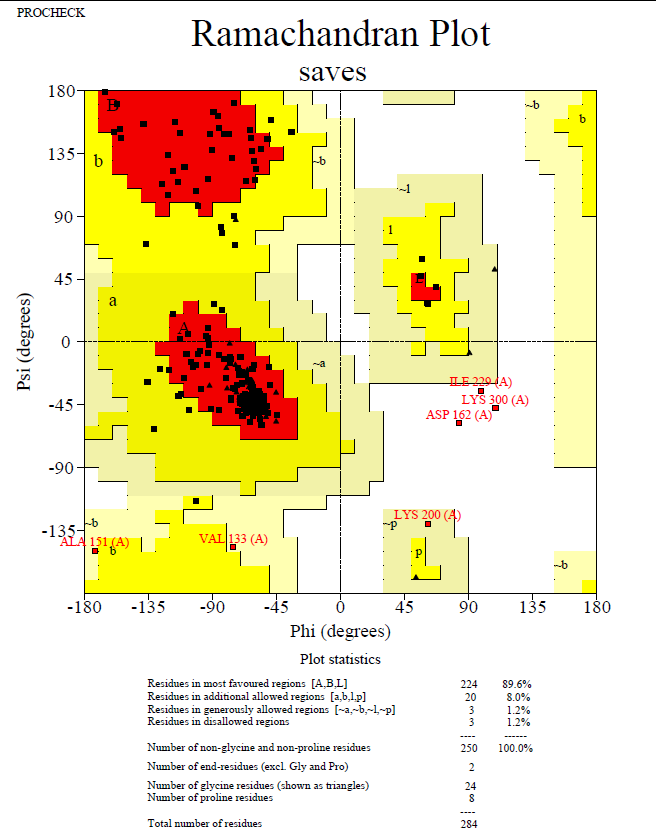
 **TcZIP10 TcZIP11**

**Supplementary Figure 1**. Evaluation of all approved protein models. Ramachandran plot generated by PROCHECK server, validating backbone dihedral angels of the energy minimized model.
