# Supplementary material for: Zinc/iron-regulated transporter-like protein gene family in Theobroma cacao L: Characteristics, evolution, function and 3D structure analysis
Source: Front Plant Sci. 2023 Feb 28;14:1098401. doi: 10.3389/fpls.2023.1098401 (PMC10012423; doi:10.3389/fpls.2023.1098401)
Supplement: Supplementary file 2 [file DataSheet_2.docx]

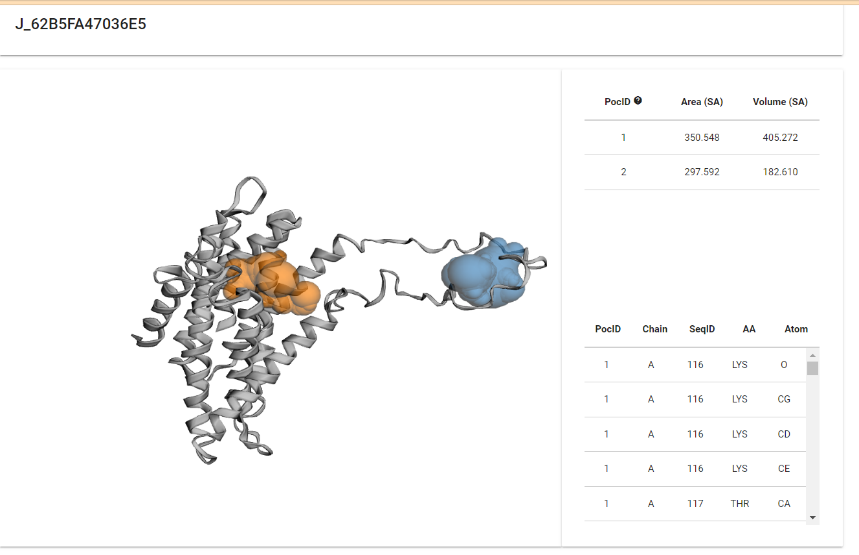
**TcZIP1 TcZIP2 TcZIP3**


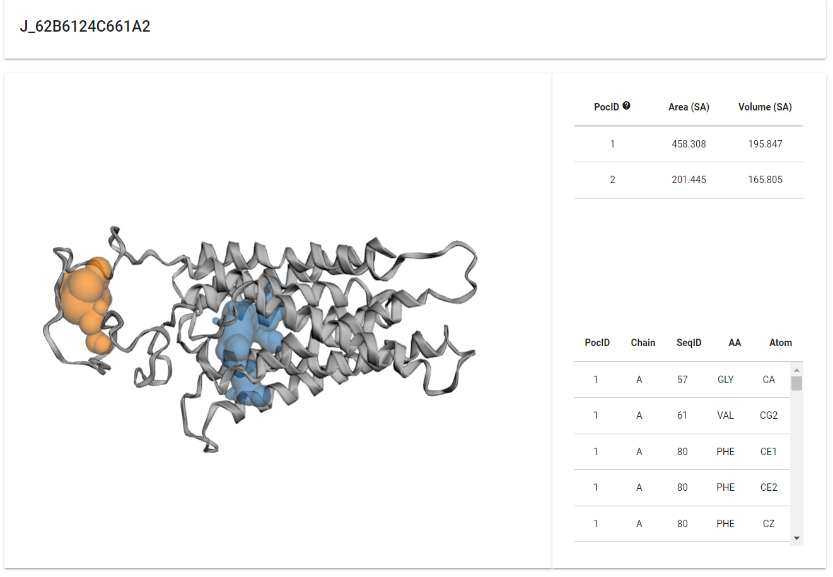

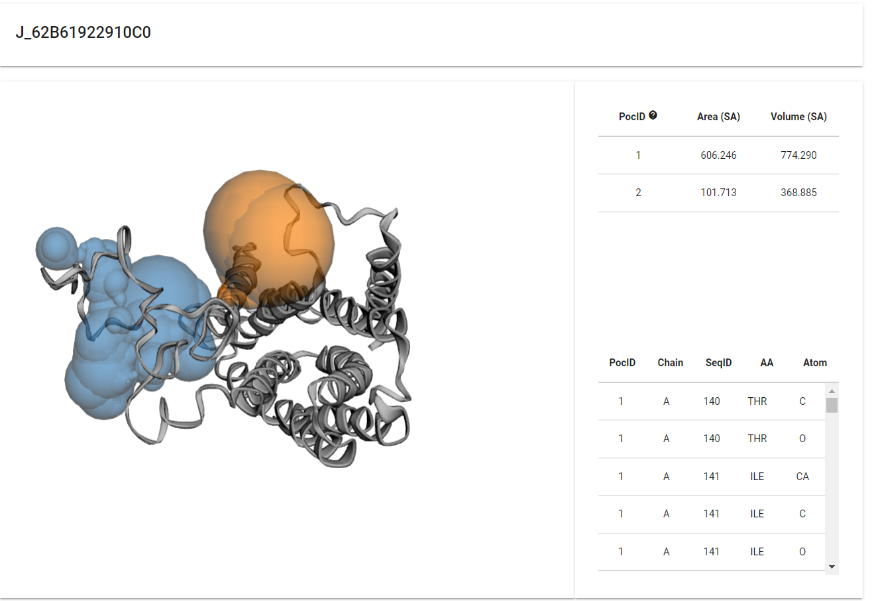


**TcZIP4 TcZIP5 TcZIP6**


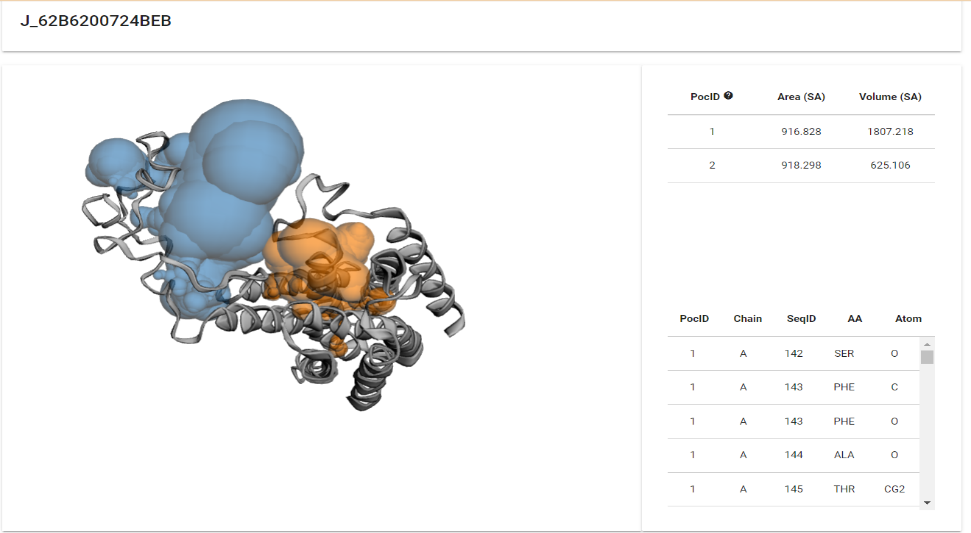

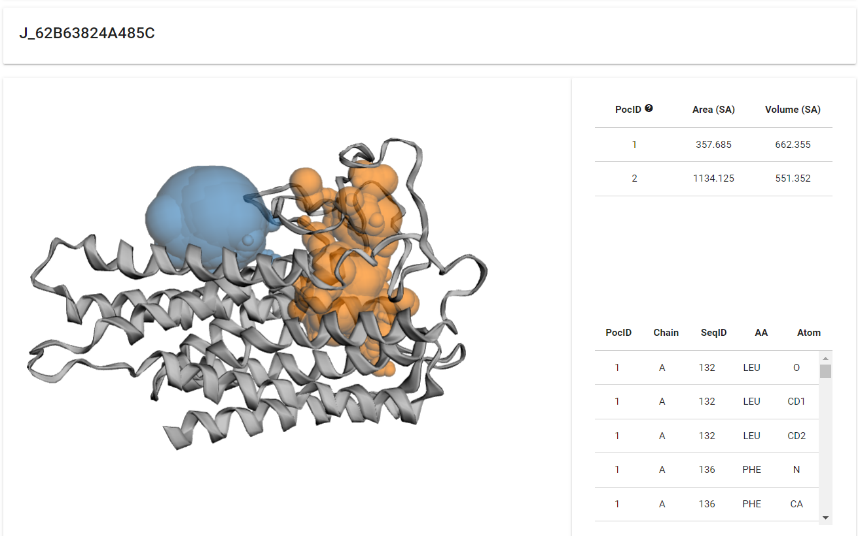

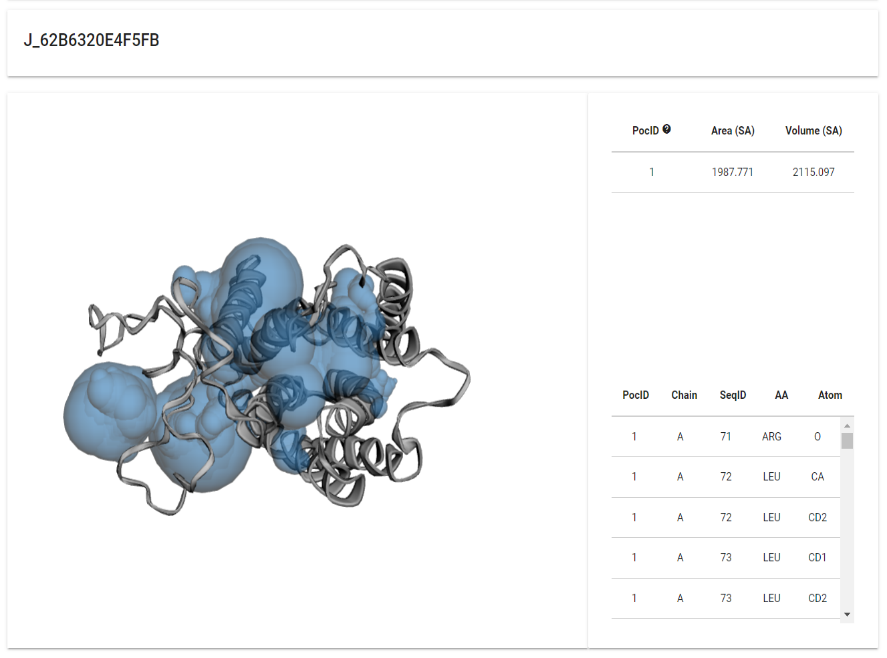


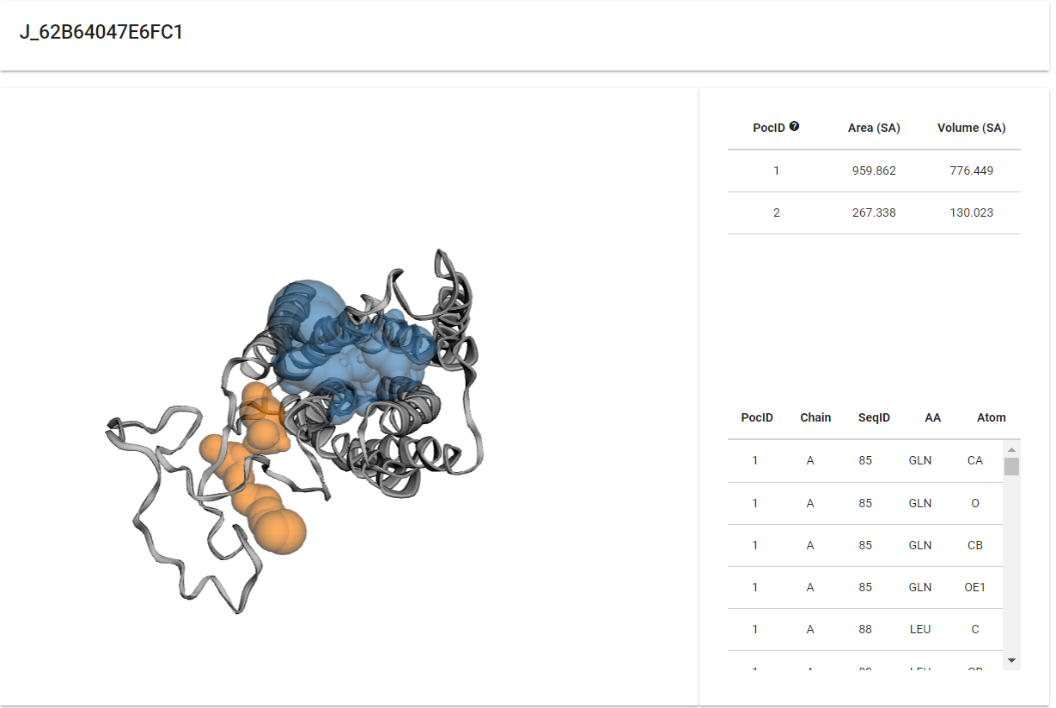
**TcZIP7 TcZIP8 TcZIP9**


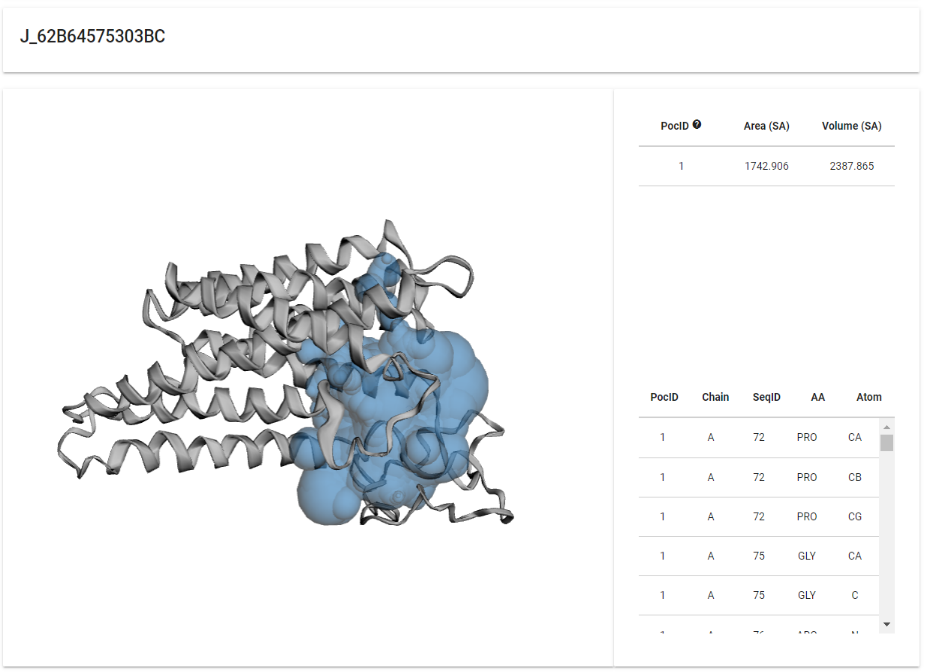

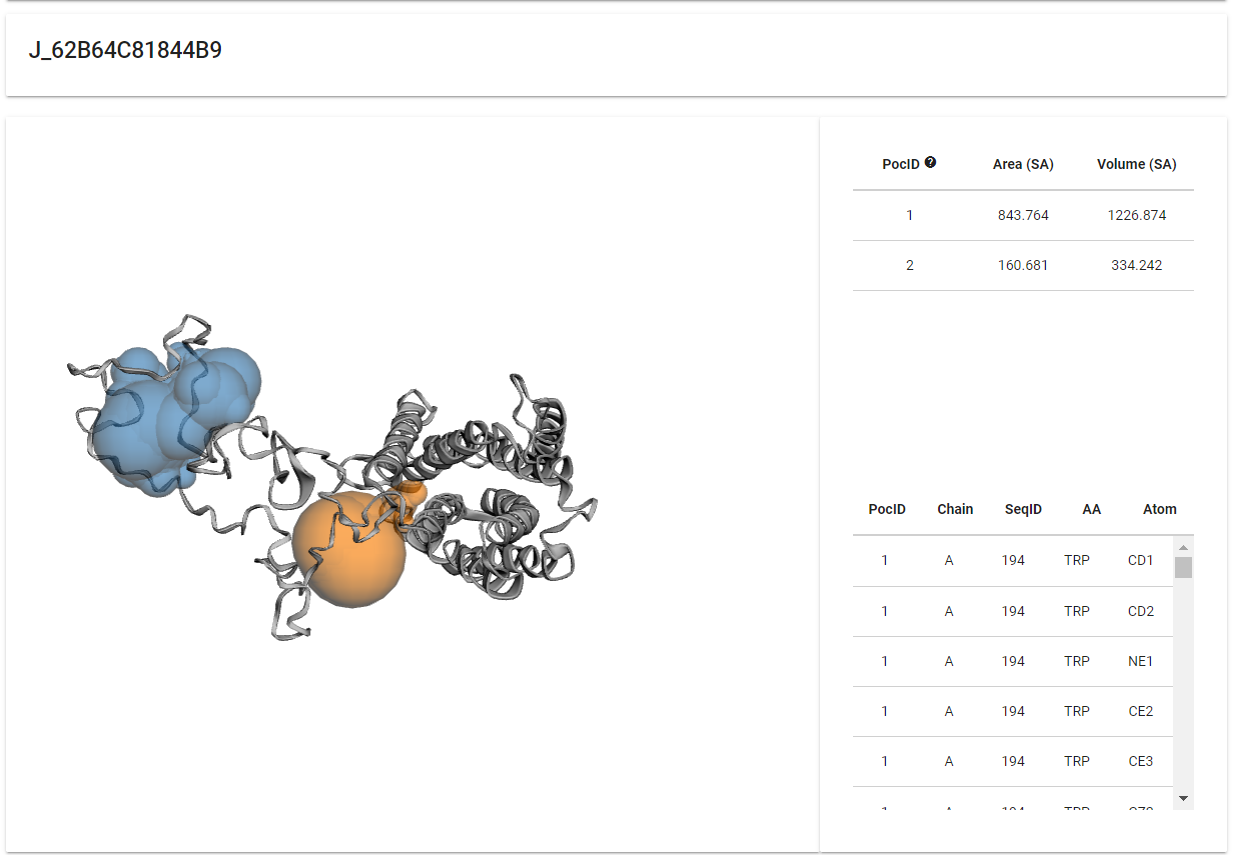


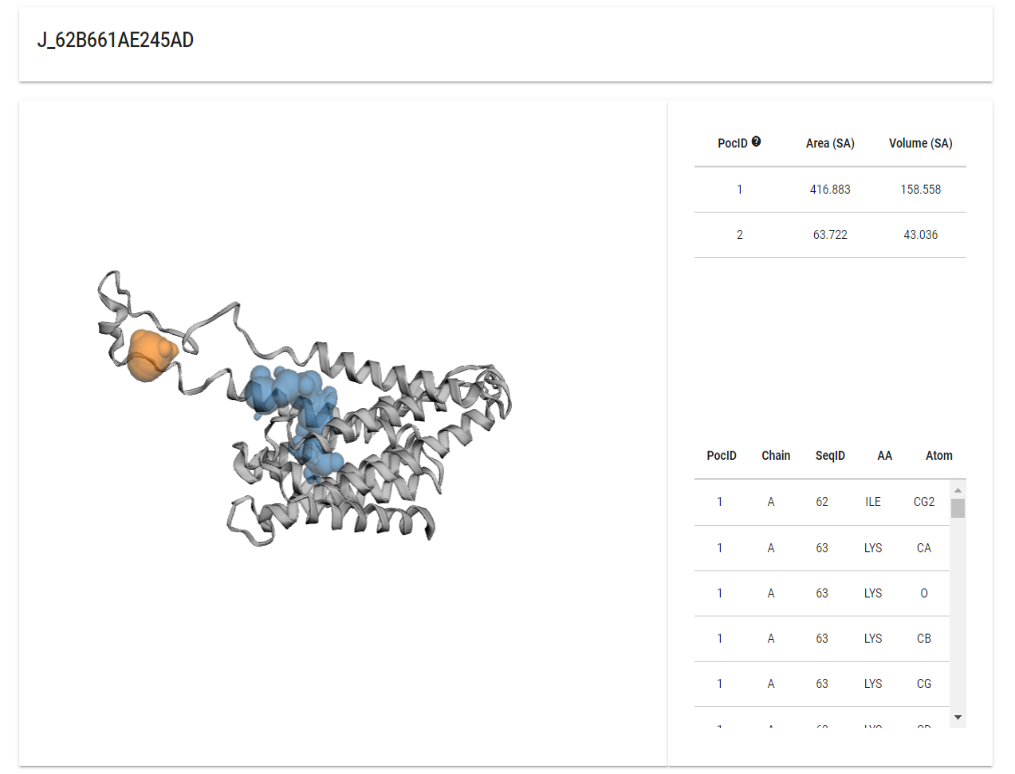

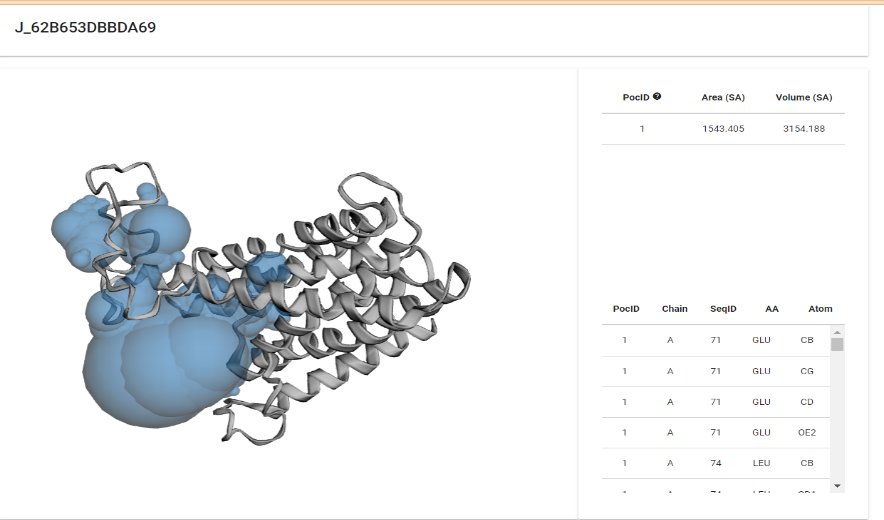
**TcZIP10 TcZIP11**

**Complementary Figure 1 -** The predicted pocket regions and metal binding sites in ZIP transport proteins in *T. cacao.*

**Supplementary Figure 2**. Predicted pocket regions in ZIP transport proteins in cocoa. The predicted 3D structures of the TcZIPs proteins were generated using the Swiss-Model server and the pockets were identified by the CASTp 3.0 server. The molecular pockets with the largest volume in the 3D structures were identified by the server, indicated in blue (the larger) and orange (the smaller) pocket sites.
