# Supplementary material for: Zinc/iron-regulated transporter-like protein gene family in Theobroma cacao L: Characteristics, evolution, function and 3D structure analysis
Source: Front Plant Sci. 2023 Feb 28;14:1098401. doi: 10.3389/fpls.2023.1098401 (PMC10012423; doi:10.3389/fpls.2023.1098401)
Supplement: Supplementary file 3 [file DataSheet_3.docx]

**Complementary Figure 3 - Percentage of amino acids distributed in the predicted pocket for the ZIP family in *T. cacao*.** For this analysis we counted only the predicted amino acids in the predicted protein pockets**.**
